# A carbonic anhydrase-based nanogel for cyanobacterial growth enhancement

**DOI:** 10.1016/j.mtbio.2025.102622

**Published:** 2025-12-01

**Authors:** Marius Stoeckle, Carmen M. Domínguez, Abbey Hanes, Simone Weigel, Alexei Kiselev, Kersten S. Rabe, Christof M. Niemeyer

**Affiliations:** a Institute for Biological Interfaces 1 (IBG1), Karlsruhe Institute of Technology (KIT), D-76344, Eggenstein-Leopoldshafen, Germany; b Institute of Meteorology and Climate Research, Department of Atmospheric Aerosol Research (IMK-AAF), KIT, D-76344, Eggenstein-Leopoldshafen, Germany

**Keywords:** Bioconjugation, Carbonic anhydrase, CO_2_ capture, Cyanobacteria, Enzyme immobilization

## Abstract

Sustainable bioproduction requires improved carbon fixation in phototrophic microorganisms. Cyanobacteria such as *Arthrospira platensis* and *Synechocystis* sp. PCC 6803 convert CO_2_ into biomass using sunlight, yet their growth under ambient conditions is often limited by low CO_2_ solubility. Here, we report the development of self-assembling all-enzyme nanogels composed of carbonic anhydrase (CA) to enhance CO_2_ bioavailability in cyanobacterial cultures. These carrier-free nanogels were formed through covalent self-assembly of CA variants fused to SpyTag or SpyCatcher domains and characterized using electrophoresis, dynamic light scattering, atomic force, environmental scanning electron, and confocal laser scanning microscopy. A custom pH-based CO_2_ hydration assay showed that the nanogels retained high catalytic activity and stability for several weeks, and systematic analyses of buffer capacity revealed CA-dependent changes in the extracellular environment. Although CA supplementation had only minor effects on *A. platensis*, *Synechocystis* cultures exhibited a clear increase in biomass and chlorophyll content. Overall, CA nanogels represent a robust, carrier-free platform for enhancing CO_2_ utilization and hold promise for applications in carbon capture, biomass production, and multi-enzyme cascade systems.

## Highlights

• Carbonic anhydrase (CA) nanogels were synthesized through covalent self-assembly.
• Nanogels were characterized and their effect on culture pH and buffering assessed.
• Nanogels stayed active for weeks in cyanobacterial cultures.
• Nanogels boosted biomass and chlorophyll levels.

## Graphical abstract

**Figure:**
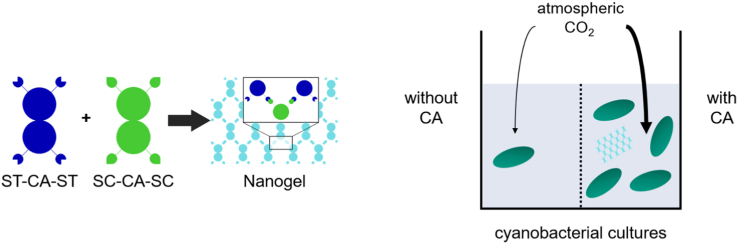

## 1 Introduction

Since the industrial revolution, coal- and petroleum-based chemical production has driven a linear economy with high CO_2_ emissions. In light of the climate crisis, more carbon-efficient synthesis routes are urgently needed [1]. To address this need, cyanobacteria present a promising solution: they use sunlight as an energy source and CO_2_ as their sole carbon input. Combined with genetic engineering, they can produce valuable compounds while capturing CO_2_ — unlike conventional methods that emit it [2]. However, the dissolution rate of atmospheric CO_2_ within an aqueous system is very poor, leading to low production and growth rates, which limit the industrial use of cyanobacteria [3]. One potential strategy to overcome this limitation is to enhance CO_2_ availability in the growth medium using carbonic anhydrase (CA) enzymes. These zinc-containing metalloenzymes catalyze the rapid conversion of CO_2_ into bicarbonate by facilitating a nucleophilic attack via a zinc-bound hydroxyl group [4].

Notably, some CAs exhibit exceptionally high turnover rates and remain active at temperatures exceeding 80 °C, making them particularly attractive for industrial applications, for example in the direct capture of flue gas [5,6]. Additionally, thermostability is commonly associated with prolonged enzyme activity, making thermostable enzymes also attractive candidates for long-term use in cyanobacterial cultures. In such systems, enzymes must withstand challenges such as secreted proteases, potential inhibitors like chelators, and physical stress. Immobilizing CAs on support materials or utilizing a surface-display approach [7] has been shown to mitigate these effects by creating a protective microenvironment that shields the enzyme from degradation and extends its functional lifespan [[8], [9], [10]].

While enzyme immobilization is typically performed on solid carriers, this approach often suffers from drawbacks such as limited biocompatibility and reduction of enzymatic activity due to non-directional immobilization. These limitations can be overcome by our recently developed self-assembling all-enzyme hydrogels (AEHs) [11]. Composed entirely of enzymes, AEHs are formed through the efficient self-assembly of genetically encoded SpyTag (ST) and SpyCatcher (SC) domains [12] fused to arbitrary enzymes of interest [[13], [14], [15], [16], [17], [18], [19]]. While the assembly of complementarily modified enzyme building blocks in solution initially results in the formation of nanogels [11], increasing the concentration through dehydration enables the formation of cohesive hydrogel structures and even porous foam materials [19]. These biocatalytically active materials exhibit outstanding stability in microfluidic reactors, enabling continuous operation over several days to weeks.

Given the excellent performance of AEH materials, we here report on their application for producing carbonic anhydrase nanogels using the thermostable CA from *Sulfurihydrogenibium azorense* [5], aiming to explore their utility in cyanobacterial cultures. To this end, we investigated the impact of CA on two cyanobacterial species: *Synechocystis* sp. PCC 6803 (abbreviated in the following as *Synechocystis*) and *Arthrospira platensis* (commonly known as Spirulina). *Synechocystis* is a well-established model organism and was the first phototroph to have its genome fully sequenced, which has enabled extensive genetic engineering for applications such as terpene biosynthesis [20,21]. Its relatively slow growth makes it a suitable target for evaluating improvements in carbon supply [22], with potential implications for other slow-growing phototrophs. *A. platensis*, on the other hand, is widely cultivated as an industrial food supplement due to its rich composition of essential amino acids, polyunsaturated fatty acids, and minerals, making it highly relevant for large-scale applications [23].

CA-based all-enzyme nanogels were first characterized by electrophoresis, dynamic light scattering, and atomic force and electron microscopy, and subsequently applied as additives in *Synechocystis* and *A. platensis* cultures. Fluorescently labeled enzyme variants further enabled visualization of their localization within the cyanobacterial cells. The presence of CA nanogels significantly enhanced CO_2_ dissolution, increasing its bioavailability to the cyanobacteria and thereby promoting improved growth. These results highlight the potential of enzyme-based materials to optimize carbon supply in phototrophic cultivation systems.

## 2 Materials and methods

### 2.1 Construction of plasmids

Plasmids were linearized using a PCR reaction. The primers used in this study are listed in Table 1. To allow a successful recombination in the next steps, primers and gene fragments were designed to have a sequence overlap of 20–30 base pairs. Genetic constructions of the expression vectors were carried out using isothermal recombination described by Gibson et al. [24]. The reaction was carried out at 50 °C and 600 rpm for 1 h. Then, the reaction mixtures were treated with DpnI (New England Biolabs) to remove any remaining DNA from prior PCR reactions. The resulting plasmids were transformed into chemically competent *E. coli* DH5α cells. Plasmids were purified using a ZR Plasmid Miniprep Classic Kit (Zymo Research) according to the manufacturer’s instructions. Sequences were verified by commercial sanger sequencing (LGC Genomics). eGFP-tagged variants and SC were prepared and purified as described previously [25].

**Table 1:** Name and sequence of the used primers.

Name	Sequence
MS73	CTCCAATGATGAACTTCTGCTGAACCGCCACCACCAATATG
MS74	CTGGAAAGCAACTAATGAGATCCGGCTGCTAACAAAGCCCG
MS75	CTCCAATGATGAACTTCTGCGCTGCCACCACCACCTTTGG
MS132	ATGGTTGATGCCTATAAACCGACCAAATAATGAGATCCG GCTGCTAACAAAGCCCGAAAG
MS133	CGGTTTATAGGCATCAACCATAACAATATGTGC GCTGCCACCACCACCGTTGCTTTCCAGAATATAAC
MS146	GAATATAACGGCTATTGATCGG
MS147	CTAACAAAGCCCGAAAGGAAGC
KSR65rev	ATGTATATCTCCTTCTTAAAGTTAAAC
MP49	ATTACCCTGGGTATGGATGAACTGTATAAAGGTGGTGGTGGTAGCGCAC

**pET-22b+_ST-CA-ST**: A ST-containing pET-22b + plasmid was linearized by PCR using the primers MS74 and MS75. This DNA backbone was then used in a recombinant Gibson assembly with an insert encoding the CA gene (UniProt accession number: ACN99362.1). The insert was ordered as a codon-optimized DNA fragment from GeneArt Gene Synthesis (Thermo Fisher Scientific). Subsequently, the resulting plasmid was employed in a second PCR reaction. The primers MS132 and MS133 inserted the second ST, leading to a N-terminal 6xHis-tag + ST and a C-terminal ST, all linked to the CA gene by a GGGS-linker.

**pET-22b+_SC-CA-SC**: A SC-containing pET-22b + plasmid was linearized by PCR using the primers MS73 and MS74. This DNA backbone was then used in a recombinant Gibson assembly with an insert encoding the CA gene. The insert was ordered as a codon-optimized DNA fragment from GeneArt Gene Synthesis (Thermo Fisher Scientific). The resulting plasmid was again linearized in a PCR reaction using the primers MS146 and MS147. Another gene fragment encoding a second SC was used in the Gibson assembly in order to insert the C-terminal SC. The final sequence consisted of a N-terminal 6xHis-tag + SC and a C-terminal SC, all linked to the CA gene by a GGGS-linker.

**pET-22b+_ST-eGFP-ST**: A ST-containing pET-22b + plasmid was linearized by PCR using the primers KSR65rev and MP49. This DNA backbone was then used in a recombinant Gibson assembly with an insert encoding the ST-eGFP gene. The final sequence consisted of a N-terminal ST and a C-terminal 6xHis-tag + ST, all linked to the eGFP gene by a GGGS-linker.

### 2.2 Protein expression

For heterologous expression of the CA variants, *E. coli* BL21(DE) cells were transformed with the respective plasmids. Transformed cells were plated on LB agar plates containing ampicillin (final concentration: 0.1 mg mL^−1^) and incubated overnight at 37 °C. On the following day, single colonies were used to inoculate 50 mL of liquid TB_Amp_. The cultures were grown overnight at 37 °C and 180 rpm. For large-scale expression, 40 mL of the overnight culture were transferred into 2 L of fresh TB_Amp_ and incubated at 37 °C and 180 rpm until an OD_600_ of 0.4–0.8 was reached. Protein expression was induced by the addition of IPTG to a final concentration of 1 mM for 5 h at 37 °C. Cells were harvested by centrifugation (10,000×*g*, 10 min), resuspended in Buffer A (50 mM sodium phosphate, 300 mM NaCl, 10 mM imidazole, pH 8.0), and stored at −80 °C until further use.

### 2.3 Protein purification

Frozen cells were thawed and treated with DNaseI and lysozyme (AppliChem) for 30 min on a rotary mixer at room temperature. Cell disruption was performed via ultrasonication, followed by centrifugation at 45,000×*g* for 1 h at 4 °C. The supernatant was filtered through a 0.45 μm Durapore PVDF membrane (Steriflip, Millipore). To purify the protein, the clear lysate was loaded on a 5 mL His60 Ni Superflow Cartridge (Clontech) mounted on an Äkta Pure liquid chromatography system (GE Healthcare). The column was equilibrated, washed with Buffer A and then, the bound proteins were eluted with Buffer B (Buffer A supplemented with 500 mM imidazole). The eluted protein was buffer-exchanged to 100 mM Tris-HCl with 100 mM NaCl (pH 8.0) using Vivaspin® centrifugal concentrators (Sartorius). Protein samples were flash-frozen in liquid nitrogen and stored at −80 °C.

### 2.4 Sodium dodecyl sulfate–polyacrylamide gel electrophoresis (SDS-PAGE) analysis

Protein samples were mixed with 4 × SDS-PAGE loading buffer (500 mM Tris-HCl, 20 % (v/v) glycerin, 0.4 % (w/v) SDS, 5 % (v/v) β-mercaptoethanol, 1.9 mg mL^−1^ bromophenol blue, pH 6.8), boiled at 95 °C for at least 5 min, and loaded onto 16 % PA gels or 4–15 % Mini-PROTEAN® TGX™ precast protein gels (Bio-Rad). Electrophoresis was carried out at a constant current of 55 mA for 30 min. Gels were stained with Coomassie Brilliant Blue R-250 for 15 min and destained in water. The PageRuler® Prestained Protein Ladder (Thermo Fisher Scientific) was used for size estimation. The visibility of the bands was improved using the software of the Vilber Fusion FX gel documentation device.

### 2.5 Protein immobilization and conjugation assay

Nanogel formation was initiated by mixing equimolar solutions of SC-CA-SC and ST-CA-ST. Subsequently, the mixture was incubated on a thermoshaker at 30 °C and 600 rpm for 2 h. To analyze the binding kinetics during polymerization, samples were taken at defined time points, mixed with 4 × SDS-PAGE loading buffer, and boiled for at least 5 min to terminate the reaction. Samples were subsequently analyzed via SDS-PAGE. Immobilization of SC on Dynabeads M − 270 Epoxy beads (Thermo Fisher Scientific) was achieved, following the manufacturer’s instructions. Subsequently, either ST-CA-ST or pre-assembled nanogels were incubated together with the beads for 30 min at 30 °C.

### 2.6 Dynamic light scattering

100 μL of a 1000 μM protein solution was transferred into an UV-transparent cuvette. Measurements were performed using a Nano-Series ZetaSizer Nano ZSP (Malvern Instruments) equipped with a He−Ne-Laser (633 nm). The system was set to 30 °C. The hydrodynamic radius (Z-Average) was recorded over a period of 2 h.

### 2.7 Atomic force microscopy

For AFM characterization, 13 nM nanogel samples were prepared in 100 mM Tris-HCl buffer supplemented with 100 mM NaCl. Then, 10 μL of this solution were deposited onto freshly cleaved mica (Plano GmbH). After 10 min incubation at RT, 70 μL of Tris-100 mM NaCl buffer were added. Samples were imaged using a NanoWizard 3 (JPK) atomic force microscope in tapping mode with pyramidal tips (SNL-10, radius 2 nm, spring constant 0.35 N/m, Bruker). Images were processed using the JPK data processing software (polynomial subtraction level 1, gaussian smoothing 500 mpx). The processed images were subsequently analyzed with ImageJ to determine the particle size distribution. This involved converting the images to binary format and measuring the Feret diameter of the individual particles.

### 2.8 Electron microscopy

The nanogel samples were analyzed with Environmental Scanning Electron Microscope (ESEM, ThermoFisher FEI Quattro S), using variable beam acceleration voltage between 5 kV and 20 kV under vacuum conditions. Prior to scanning, the buffer of the nanogel was exchanged to ddH_2_O and the samples were dried on a cover slide. The samples were coated with 1.5 nm Pt to ensure electric conductivity.

### 2.9 Fluorescence microscopy

Cyanobacterial cultures were cultivated for two weeks in the presence of 1.25 μM CA and GFP proteins. To visualize the localization of the proteins on the cyanobacterial cells, equal amounts of either the CA nanogel or the correspondingly tagged CA enzymes (ST-CA-ST coupled to SC-eGFP, or SC-CA-SC coupled to ST-eGFP) were used. In the case of the CA nanogel, equal amounts of 400 μM ST-eGFP-ST were added during the assembly process. Samples were collected after 2 h or two weeks, diluted 1:1 with water, and fixed at 65 °C for 10 min. Fluorescence microscopy was performed using an LSM 880 microscope operated in Airyscan mode. Image acquisition and processing were conducted with Zen Black and Zen Blue software, respectively. eGFP fluorescence was visualized in green (excitation 488 nm, emission 516 nm), and cellular autofluorescence in red (excitation 561 nm, emission 579 nm).

### 2.10 Calculation of cell and nanogel surface areas

To estimate the potential binding capacity of the nanogel on the *Synechocystis* cell surface, the total number of cells and their corresponding surface area were first determined. An inoculation density of OD_730_ = 0.1 corresponds to 7 ∗ 10^6^ cells mL^−1^ [26], resulting in 8.4 ∗ 10^7^ cells in a 12 mL culture. Assuming an average cell diameter of 2 μm (r = 1 μm), the surface area of a single cell is 12.566 μm^2^, yielding a total cellular surface area of 1.01 ∗ 10^9^ μm^2^ for the entire culture. The nanogel particles had an average diameter of 50 nm (r = 25 nm) as determined by AFM, corresponding to a projected surface area of 0.001963 μm^2^ per particle. At a CA concentration of 1.25 μM in 12 mL, this equals 9.03 ∗ 10^15^ enzyme molecules, or approximately 9.03 ∗ 10^14^ nanogel particles when assuming ten enzyme molecules per nanogel. The combined surface area of all nanogel particles is therefore 1.77 ∗ 10^12^ μm^2^. Based on these numbers, comparison of the total nanogel surface area to the total cellular surface area indicates that only 0.057 % of the nanogel surface would be required to fully cover all *Synechocystis* cells.

### 2.11 Activity assay using p-nitrophenyl acetate and CO_2_

The degree of esterase activity was measured in a Synergy H16 plate reader at 30 °C. The reactions were initiated by the addition of p-nitrophenyl acetate (final concentration: 180 μM) to a solution containing 4 μM enzyme in reaction buffer (100 mM Tris-HCl, 100 mM NaCl, pH 8.0). Absorbance at 348 nm was recorded over a period of 60 min. The change in absorbance was calculated by subtracting the initial value from the final value. The activity of the nanogel was set to 100 %, and all other values were normalized accordingly. For biochemical characterization, enzyme activities without any supplementation were set to 100 %. To assess the impact of pH, a 100 mM Britton-Robinson buffer supplemented with 100 mM NaCl was adjusted to the desired pH and subsequently used in the assay. Salts were added to a final concentration of 7 mM, EDTA to 2 or 4 mM, and acetazolamide to 10, 20, 30, or 40 nM. For thermostability analysis, enzyme samples were incubated at 75 °C for 20 min and subsequently cooled on ice prior to the activity assay.

An alternative method for assessing enzyme activity involves the use of CO_2_ to lower the pH of a solution when it is bubbled into the solution. Real-time pH measurements were performed using a pH meter, enabling kinetic analysis [27]. To ensure reproducibility, all assays were conducted in 50 mM Tris-HCl buffer with 100 mM NaCl (pH 9), providing sufficient buffer capacity, even in media or culture supernatants. Subsequently, the initiation of the CO_2_ flow rate at 1.5 mL s^−1^ was executed, and the pH values were meticulously documented at 5-s intervals for a duration of 2 min. This procedure was conducted within 10 mL volumes, under continuous stirring. Controls without CA served as baselines. These were then compared to samples that had been supplemented with enzyme. Cyanobacterial supernatants were centrifuged beforehand to remove cells. Activity was defined as the average pH drop over 1 s during the initial reaction phase, relative to the baseline (also described in section 3). Freshly prepared enzyme samples were utilized at a final concentration of 1.25 μM. For protease treatment, enzymes were incubated overnight at 37 °C with trypsin at a final concentration of 1, 5, or 10 mg mL^−1^.

### 2.12 Cyanobacterial growth and analytical methods

*Synechocystis* cultures were obtained from the American Type Culture Collection (reference: 27184). *Arthrospira platensis* cultures (non-axenic) were obtained from the Culture Collection of Algae at the University of Göttingen in Germany. Two distinct ecotypes were utilized in this study. The specific references are: SAG 21.99 and SAG 257.80. A sample of the open pond culture of *A. platensis* was kindly provided by Acheron GmbH (Bremen, Germany). The images of the cultures were obtained using an Invitrogen™ EVOS™ XL Core Imaging system.

All cultures were grown in a translucent incubator maintained at 25 °C under atmospheric CO_2_ levels and without shaking, reflecting the low-energy conditions of unstirred open pond systems that rely on natural sunlight and passive gas exchange. A detailed description of the cultivation chamber, the passive-exchange culture tubes, and the environmental light conditions is provided in Fig. S1. The incubator was placed 50 cm from a north-facing window, which served as the sole light source and provided a natural day–night cycle (Fig. S1A). All cultures within each experiment were incubated simultaneously in the same chamber to ensure identical environmental conditions (Fig. S1A–B). Fig. S1C–E summarize the incident light characteristics relevant for cyanobacterial growth, including the spectral distribution measured with a LI-180 spectrometer (panel C) and the sunshine hours, irradiance values, and resulting estimates of photosynthetically active radiation (PAR) (panels D–E). These measurements reflect the naturally diffuse and comparatively low light intensities resulting from the seasonal conditions at the study site and the use of a north-facing window, which provides realistic illumination levels frequently encountered in open pond cultivation.

The cultivation of *Synechocystis* was performed in BG11+ medium without carbonate (0.04 g L^−1^ K_2_HPO_4_; 1.5 g L^−1^ NaNO_3_; 0.075 g L^−1^ MgSO_4_ x 7 H_2_O; 0.036 g L^−1^ CaCl_2_; 0.001 g L^−1^ EDTA; 0.006 g L^−1^ trisodium citrate; 0.006 g L^−1^ ammonium ferric citrate; 0.02 mg L^−1^ vitamin B12; 0.22 mg L^−1^ ZnSO_4_; 2.9 mg L^−1^ H_3_BO_4_; 0.05 mg L^−1^ Co(NO_3_)_2_; 0.29 mg L^−1^ Na_2_MoO_4_; 0.08 mg L^−1^ CuSO_4_). *A. platensis* medium consisted of: 0.25 g L^−1^ K_2_HPO_4_; 1.25 g L^−1^ NaNO_3_; 0.5 g L^−1^ K_2_SO_4_; 0.5 g L^−1^ NaCl; 0.1 g L^−1^ MgSO_4_ x 7 H_2_O; 0.02 g L^−1^ CaCl_2_; 0.005 g L^−1^ FeSO_4_ x 7 H_2_O; 0.044 g L^−1^ EDTA; 0.005 mg L^−1^ vitamin B12; 0.005 mg L^−1^ ZnSO_4_; 5 mg L^−1^ MnSO_4_; 0.05 mg L^−1^ H_3_BO_4_; 0.005 mg L^−1^ Co(NO_3_)_2_; 0.005 mg L^−1^ Na_2_MoO_4_; 3.5 mg L^−1^ FeSO_4_.

A volume of 12 mL of cyanobacterial culture was cultivated within a commercially available cell culture flask (Nunc™ EasYFlask 25 cm^2^ Nuclon™ Delta Surface, Thermo Fisher Scientific). The optical densities at 750, 680, 730 and 565 nm were obtained using a Synergy H16 plate reader. The pathlength correction was employed to obtain a value corrected to 1 cm pathlength. The dry weights of *Synechocystis* cultures were calculated based on these values, using a previously described formula [28].

(1) Dry cell weight [g L^−1^] = *OD*_*730*_ ∗ 0.16

The amount of chlorophyll in *Synechocystis* cultures was calculated based on a correlation previously reported by others [29] (Equation (2)). To provide the chlorophyll concentration per unit of OD_750_, the chlorophyll concentration [nmol mL^−1^] was divided by OD_750_.

(2) Chlorophyll concentration [nmol mL^−1^] = (*OD*_*680*_ – *OD*_*750*_) ∗ 10.814

Subsequently, one-third of the culture was centrifuged, the cell pellet was discarded and the supernatant was transferred back into the culture. Fresh medium was supplied in order to avoid drying out.

For the calculation of *A. platensis* dry weights equation (3) [30] was used:

(3) Dry cell weight [g L^−1^] = (*OD*_*565*_ – 0.0253) / 2.4408

In the experiments, the final CA concentration in the cyanobacterial cultures was adjusted to 1.25 μM. Each culture was initially adjusted to an optical density of 0.1 (measured at 730 nm for *Synechocystis* and at 565 nm for *A. platensis*), corresponding to a dry cell weight of 0.016 g L^−1^ and 0.03 g L^−1^, respectively. The nanogel was prepared as described above. For an initial control, 1.25 μM BSA was supplemented. After 6 weeks, the cyanobacteria were centrifuged and the supernatant was concentrated using a VivaSpin system (Sartorius) with a 5 kDa cut-off value. The remaining liquid was utilized in an SDS-PAGE to detect the remaining enzymes.

### 2.13 Determination of the buffering capacity

For determination of buffering capacity 10 mL of sample liquids were used. The initial pH value was recorded. Then, a 0.12 mol L^−1^ HCl solution was carefully titrated until the pH value dropped by 1 unit. The volume of acid was used to calculate the buffer capacity.

### 2.14 Statistical analysis

Statistical analyses were performed using one-way ANOVA in SPSS software (IBM Corp.) to evaluate differences between experimental groups. The assumption of homoscedasticity was tested using Levene’s test. Pairwise comparisons were conducted using the Tukey HSD post hoc test when homoscedasticity was met, or the Dunnett’s T3 post hoc test when it was not. A *P*-value of <0.05 was considered statistically significant. Exact *P*-values for all pairwise comparisons are provided in Table S1.

## 3 Results and discussion

### 3.1 Characterization of the nanogel

To explore the development and application of novel CA-based, biocatalytically active nanogel materials, expression plasmids were first constructed to encode variants of the thermostable CA from *Sulfurihydrogenibium azorense*. Thermostable enzymes are generally known to retain their catalytic activity for longer periods compared to their mesophilic counterparts, making the CA from *S. azorense* a promising candidate for application in cyanobacterial cultures. In addition to its stability, this enzyme exhibits exceptionally high catalytic efficiency. Its reported kcat/KM value of 3.5 ∗ 10^8^ M^−1^ s^−1^ is more than twice that of human CA (1.5 ∗ 10^8^ M^−1^ s^−1^) or the thermostable CA from *Sulfurihydrogenibium* sp. YO3AOP (1.1 ∗ 10^8^ M^−1^ s^−1^) [5]. To the best of our knowledge, the *S. azorense* CA has been described as the fastest CA since 2012, and only recently an ancestral sequence reconstruction approach identified an even faster variant [31].

**Fig. 1:**
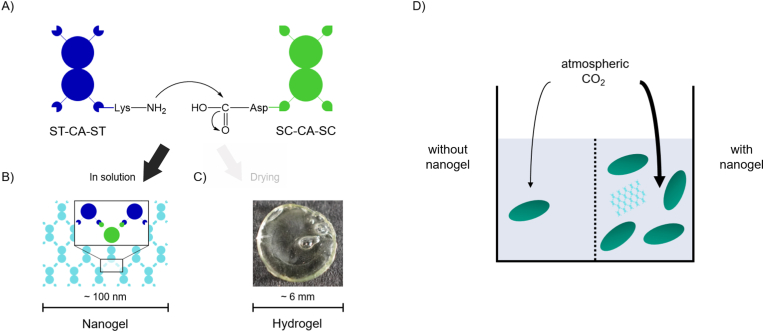
Overview of the preparation and application of carbonic anhydrase (CA) nanogels. (A) CA was genetically fused to either two SpyTag (ST) or two SpyCatcher (SC) domains, which spontaneously form covalent isopeptide bonds upon interaction. (B) Mixing stoichiometric amounts of both CA variants in buffer results in self-assembly into CA nanogels; the schematic depicts an idealized representation of the resulting supramolecular protein network. (C) Drying the enzyme mixture containing these nanogels yields a macroscopic all-enzyme hydrogel material. (D) The CA nanogels catalyze CO_2_ hydration, thereby enhancing cyanobacterial growth.

Each variant of the *S. azorense* CA was genetically fused to either a SpyTag (ST) or a SpyCatcher (SC) domain, allowing covalent nanogel formation through the spontaneous formation of an isopeptide bond between a lysine residue of the ST and an aspartic acid residue of the SC (Fig. 1A). These domains should then enable covalent self-assembly into oligomeric and polymeric structures. Due to the homodimeric quaternary structure of CA [32], each variant was designed to present either two ST or SC binding domains, respectively, ensuring the formation of a three-dimensional enzyme network (Fig. 1B). Specifically, the SC-CA-SC and ST-CA-ST variants were designed with SC or ST domains fused to both the N- and C-termini of the CA enzyme, enabling bidirectional crosslinking for network formation. The arrangement of complementarily modified enzyme building blocks in solution initially leads to the formation of nanogels, which are typically dried into solid, monolithic hydrogel pieces to enable flow biocatalysis in continuous reactors (Fig. 1C) [11]. In this study, however, we chose to retain the preparation of AEH materials at the nanogel stage by incubating the complementarily modified CA building blocks in buffer for only 2 h. This approach was intended to produce CA materials that remain in solution and can be directly added to cyanobacterial cultures, with the aim of increasing CO_2_ availability and thereby enhancing cyanobacterial growth (Fig. 1D).

Subsequent to overexpression in *E. coli* and Ni-NTA purification, the recombinant CA variants were obtained in near homogeneous purity, as judged from SDS-PAGE analysis (Fig. S2). The ST-CA-ST variant was produced at over 10 mg L^−1^, consistent with previously reported yields of 13 mg L^−1^ for unmodified CA [33]. The SC-CA-SC variant achieved even higher levels, exceeding 40 mg L^−1^, comparable to yields reported for CA fused to a chitin-binding domain [34]. Fusion tags are well known to enhance protein yield. However, predicting how a genetic fusion will affect an enzyme’s activity remains challenging, and rationalizing the outcome afterward is often equally difficult. In practice, both increases and decreases in activity have been reported, typically associated with alterations in the protein’s tertiary structure [[35], [36], [37]]. For instance, the maltose-binding protein (MBP) is commonly employed to facilitate purification. In the present case, the observed increase in SC-CA-SC yield may stem from improved solubility and more efficient translation initiation, two factors known to enhance protein expression [38].

**Fig. 2:**
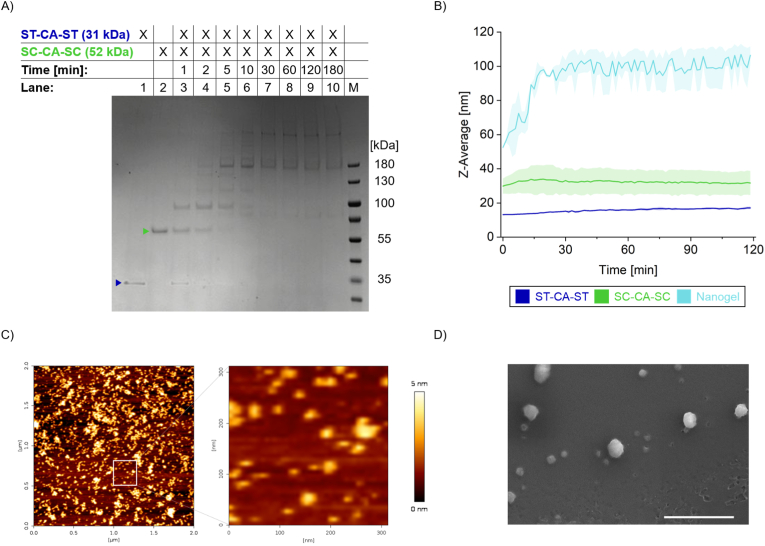
Characterization of CA nanogel materials. (A) Conjugation of ST-CA-ST and SC-CA-SC analyzed by SDS-PAGE. The enzyme variants were either applied individually (lane 1: ST-CA-ST and lane 2: SC-CA-SC) or as mixed samples (lanes 3–10). After defined time intervals, the reaction was stopped by boiling the sample in SDS loading dye. (B) Hydrodynamic diameter (Z-Average) was determined by dynamic light scattering. ST-CA-ST (blue) and SC-CA-SC (green) were measured separately or together (turquoise). Error bars represent the standard deviation from two independent experiments. (C) Atomic force microscopy (AFM) images of CA nanogels. The right panel shows a magnified view of the region indicated in the left panel. The corresponding particle size distribution is shown in Fig. S3. No particles were detected on bare mica (data not shown). (D) Representative electron microscopy image of the spherical nanogel particles. Scale bar: 1 μm.

To initially assess the binding capacity of ST-CA-ST and SC-CA-SC, stoichiometric mixtures were incubated and analyzed over time via SDS-PAGE (Fig. 2A). Within 2 min, unbound proteins began to disappear as conjugate bands appeared. By 5 min, only conjugated forms were detectable. Due to the presence of two binding domains on each enzyme, network formation likely continues until all sites are occupied or sterically hindered. After 30 min, the dominance of trimers and higher-order conjugates indicated successful nanogel formation. The calculated molecular weights are 28 kDa for untagged CA, 1.5 kDa for ST, and 12 kDa for SC. Accordingly, SC-CA-SC is expected to migrate at 52 kDa but appears at approximately 60 kDa, while ST-CA-ST has a theoretical molecular weight of 31 kDa yet migrates at around 35 kDa. Both variants therefore show a discrepancy between their calculated and apparent molecular weights. Such shifts may result from bound metal ions [39], and similar behavior has been reported for SC fusion proteins and their conjugates, which often migrate at higher apparent molecular weights than predicted [40]. This phenomenon is likely attributable to the amino acid composition of SC, which contains more acidic (19) than basic (12) residues, thereby reducing SDS binding efficiency and decreasing the effective negative charge [41,42]. The effect is further enhanced in SC-CA-SC, which contains two SC domains.

While SDS-PAGE confirmed nanogel formation, dynamic light scattering (DLS) was used to monitor particle growth over time. Stoichiometric amounts of SC-CA-SC and ST-CA-ST were mixed in a microcuvette, and the hydrodynamic radius (Z-Average) was measured over 2 h (Fig. 2B). Control measurements of the individual variants showed no change in Z-Average, confirming that particle growth resulted from specific ST/SC interactions rather than nonspecific aggregation. The nanogels reached their maximum size of ∼100 nm within 30 min, consistent with SDS-PAGE results showing only trimers and higher-order conjugates at that time. These findings align with previous AEH studies reporting a Z-Average of ∼65 nm [11].

Complementary size characterization by atomic force microscopy (AFM) provided morphological insights into the topology of the CA nanogels and served as an independent, imaging-based validation of their dimensions (Fig. 2C and Fig. S3). The AFM micrographs clearly revealed their particulate structure, and quantitative image analysis yielded a mode diameter of approximately 50 nm (Fig. S3), consistent with the DLS measurements (Fig. 2B). To further examine the morphology at high spatial resolution under near-native conditions, environmental scanning electron microscopy (ESEM) was employed. The ESEM images revealed characteristic spherical particles representing the native nanogel morphology (Fig. 2D), with dimensions in good agreement with AFM and DLS data (Fig. S4).

### 3.2 Localization of CA in cyanobacterial cultures

To investigate the potential applicability of the CA nanogels in cyanobacterial cultures and to assess whether their particulate nature influences localization, we performed high-resolution fluorescence microscopy. The ST/SC system enables facile attachment of functional proteins, such as fluorescent markers, to the CA variants. Accordingly, all enzyme variants were fused to eGFP. Since only monomeric ST-eGFP or SC-eGFP constructs were used, the formation of unintended nanogels could be excluded. In contrast, for the CA nanogel, polymerization was intended, and therefore ST-eGFP-ST was employed to ensure continuous crosslinking. The resulting protein conjugates were added directly to cultures of *Synechocystis* and *A. platensis*. Two ecotypes of *A. platensis* were analyzed to capture strain-specific differences: SAG 21.99 with its characteristic spiral morphology and SAG 257.80, which exhibits a more linear, filamentous form (Fig. S5).

**Fig. 3:**
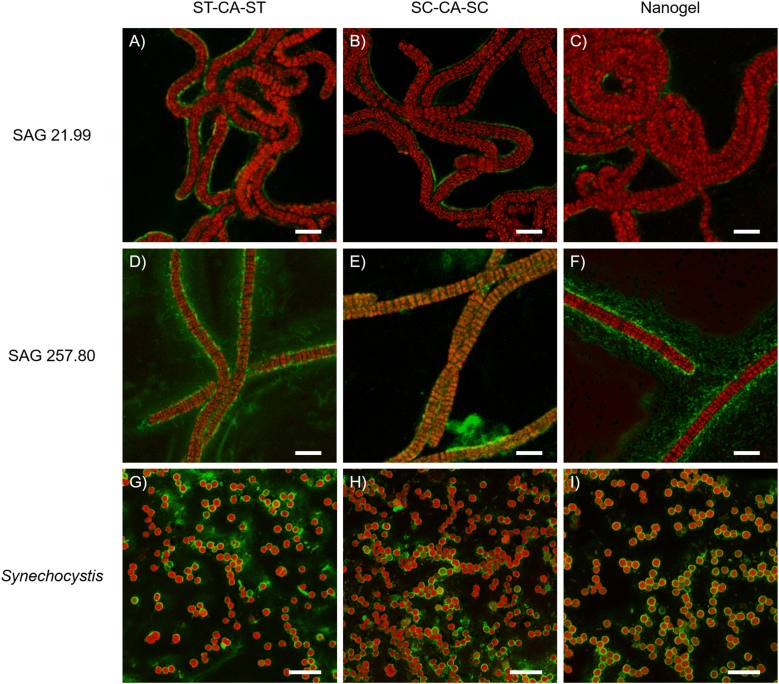
Representative fluorescence microscopy images showing the distribution of different CA formulations in cyanobacterial cultures. (A–C) *A. platensis* SAG 21.99, (D–F) *A. platensis* SAG 257.80, and (G–I) *Synechocystis* cultures were cultivated for two weeks in the presence of either individual protein constructs that do not form nanogels, ST-CA-ST coupled to SC-eGFP (A, D, G) and SC-CA-SC coupled to ST-eGFP (B, E, H), or a CA nanogel composed of ST-CA-ST and SC-CA-SC labeled with the fluorescent fusion protein ST-eGFP-ST (C, F, I). eGFP fluorescence is shown in green (excitation 488 nm, emission 516 nm), and cellular autofluorescence in red (excitation 561 nm, emission 579 nm). Scale bar: 10 μm.

After two weeks of cultivation in the presence of the CA–eGFP constructs, samples were collected and analyzed by fluorescence microscopy (Fig. 3). Autofluorescence of the photosynthetic pigments allowed visualization of the cyanobacterial cells (red channel), while eGFP fluorescence indicated the distribution of the CA variants. Control experiments with and without eGFP confirmed that the observed signal originated from eGFP (Fig. S6–S8).

For *A. platensis* SAG 21.99, both unbound enzyme variants and nanogels displayed a similar fluorescence distribution, suggesting comparable interaction behavior (Fig. 3A–C). All constructs were mainly associated with the cells, indicating a pronounced affinity toward the cell surface. In contrast, *A. platensis* SAG 257.80 showed distinct patterns: the unbound enzymes appeared as a diffuse, cloud-like signal (Fig. 3D and E), while the nanogels formed defined aggregates (Fig. 3F), reflecting clustering through ST/SC interactions. Additional fluorescence around the cell periphery suggested somewhat weaker binding in this ecotype. Comparison with samples analyzed after 2 h (Fig. S9) revealed a marked reduction of background fluorescence, indicating progressive aggregation and stabilization of nanogel–cell complexes during cultivation.

In *Synechocystis*, both unbound enzymes and nanogels predominantly associated with the cells, although diffuse fluorescence was still observed for the unbound variants (Fig. 3G and H). The nanogel appeared mainly attached to the cell surface with only minor aggregates visible in the background (Fig. 3I).

Surface display systems have previously been demonstrated in *Synechocystis* [43], while comparable strategies are not yet established in *A. platensis*. In other microorganisms, cell surface display using the SC/ST system has been achieved successfully [44,45], but these approaches require genetic modification of the host. In contrast, our fluorescence microscopy data demonstrate that the CA nanogels exhibit an intrinsic affinity toward *A. platensis* and *Synechocystis* cells, enabling spontaneous surface association without the need for engineered display systems. This represents a key advantage for industrial applications, particularly since *A. platensis* is widely cultivated as a food supplement and genetic modification is undesirable from a regulatory and consumer perspective.

A theoretical comparison between the estimated total nanogel surface area and the total *Synechocystis* cell surface area indicated that only 0.057 % of the available nanogel surface would suffice to cover all cells. However, multilayer attachment is likely, as nanogels can continue to polymerize in the cellular environment, consistent with the aggregated appearance observed in *A. platensis* SAG 257.80 (Fig. 3F). The persistence of fluorescence in all samples suggests that the CA constructs remained stable and were not completely degraded by secreted proteases, which motivated subsequent biochemical characterization.

### 3.3 Biochemical characterization of CA activities

SDS-PAGE and DLS analyses confirmed the successful formation of nanogels via the ST/SC system, a strategy expected to improve enzyme stability under the demanding conditions of cyanobacterial cultures. Before application, however, it was essential to evaluate whether nanogel formation affects enzymatic activity. To this end, two assays were performed: one based on the esterase activity of CA, and the other on its CO_2_ hydration function. Esterase activity was measured using *p*-nitrophenyl acetate (p-NPA), as product formation can be conveniently monitored at 348 nm [46]. The results showed that SC-CA-SC exhibited activity comparable to the nanogel sample, while ST-CA-ST displayed a slight reduction (Fig. S10). This suggests that nanogel formation may compensate for the reduced activity of ST-CA-ST, consistent with observations from previous studies with other enzymes [11].

**Fig. 4:**
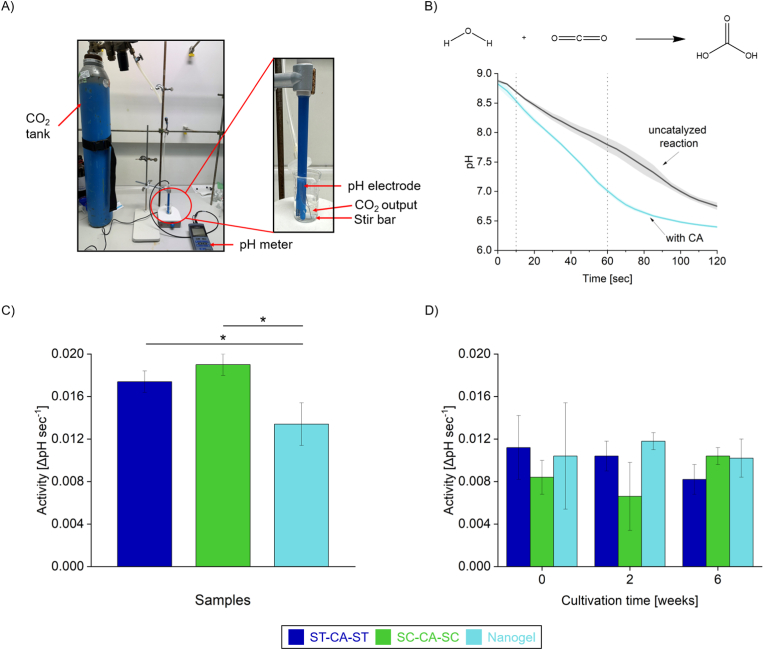
Activity assessment of CA-based catalysts. **(**A) Experimental setup for CA activity determination. The real-time measurement of pH was started when CO_2_ was bubbled into the solution. (B) Reaction scheme of CO_2_ hydration. The graph below shows exemplary measurement curves over the test duration of 2 min. The pH value drops at different rates depending on whether CA is present (here as nanogel in turquoise) or not (grey). Activity is defined as the average pH decrease over 1-s intervals, relative to the reaction without enzyme. This activity is calculated within the time window of 10–60 s. (C) Activity of freshly prepared CA variants at a concentration of 1.25 μM in pure Tris-HCl buffer, with ST-CA-ST shown in blue, SC-CA-SC in green and the nanogel in turquoise. (D) The activity of CA variants in the culture medium of *Synechocystis* sp. PCC 6803 was evaluated using either fresh medium containing the enzymes (0 weeks) or culture supernatant samples after 2 or 6 weeks of cultivation. 1.25 μM CA was initially added to the medium. Reported activities are given relative to the media without CA supplementation. To ensure accurate measurements and enable comparison with the data shown in panel (C), Tris-HCl buffer was added to all medium samples to standardize pH buffering capacity (see text for details). Error bars in panels (B), (C), and (D) indicate the standard deviation from at least two independent experiments. Statistical significance was assessed via one-way ANOVA followed by the specific post hoc test for multiple comparisons indicated in Table S1 at each tested condition in (C) and (D). Comparisons were made only among the three CA variants within each group, and not between different groups. *P*-values are indicated in Table S1. ∗*P* < 0.05.

Since the focus of this study was on CA-catalyzed CO_2_ hydration and its impact on cyanobacterial growth, an alternative assay was developed based on a previously published method [27]. This assay relies on the CO_2_-induced drop in pH, monitored in real time using a pH probe (Fig. 4A). To perform the assay, CO_2_ was introduced into the solution as a gas stream (1.5 mL s^−1^), and pH measurements were recorded for 2 min, starting simultaneously with CO_2_ flow. As CO_2_ reacts with water to form carbonic acid, the pH of the solution decreases. In the presence of CA, this reaction is catalyzed, resulting in a more rapid drop in pH (Fig. 4B). To compare CA variant performance, activity was defined as the average pH difference between catalyzed and uncatalyzed reactions during the initial 10–60 s. Freshly prepared enzymes were first tested in 50 mM Tris-HCl buffer using the CO_2_ hydration assay. While nanogel samples showed higher esterase activity with p-NPA (Fig. S10), their CO_2_ hydration activity was slightly lower than that of the individual ST-CA-ST and SC-CA-SC variants (Fig. 4C). The observed difference in activity is likely due to substrate accessibility. While p-NPA is fully dissolved and diffuses easily into the nanogels, CO_2_ must first transfer from the gas phase, dissolve in the liquid, and then diffuse into the gel matrix, thus, introducing multiple mass transfer barriers that limit substrate availability, particularly for enzymes embedded within the gels. Furthermore, differences in substrate concentration may also play a role: p-NPA was used at 32.6 mg L^−1^, while CO_2_ has a solubility of about 1.7 g L^−1^ at room temperature [47]. Since p-NPA is present at roughly 50 times lower concentration and has a lower turnover rate compared to CO_2_ hydration, any diffusion limitations caused by the nanogel are likely to be minimal.

The high-throughput nature of the esterase assay enabled a detailed biochemical characterization of the different CA formulations. To examine the influence of ionic strength, various salts were added; however, no difference in activity between the nanogel and the free enzymes was observed (Fig. S11A). Similarly, no difference in thermostability was detected after heat treatment at 75 °C for 20 min (Fig. S11B).

Since CA activity depends on the presence of a catalytically active zinc center, we next examined the effects of chelation by performing the assay in the presence of 2 mM and 4 mM EDTA (Fig. S11C). Under these conditions, the nanogel formulation displayed increased EDTA tolerance. This finding is of practical relevance, as chelating agents such as EDTA or citrate are commonly present in cyanobacterial culture media. Furthermore, the tolerance toward the well-known CA inhibitor acetazolamide was investigated [48]. At 30 nM, the nanogel formulation retained significantly higher activity than both free enzymes, and at 40 nM, significantly higher activity than ST-CA-ST (Fig. S11D).

Because cyanobacteria typically thrive in alkaline environments, we also assessed enzyme activity across a range of pH values (Fig. S11E). ST-CA-ST exhibited reduced activity at pH 6. At pH values above 8, spontaneous hydrolysis of p-NPA became pronounced, preventing reliable detection of enzymatic activity under the standard assay conditions. This limitation highlights the importance of complementary assays for activity determination.

Although nanogel formation did not enhance CA activity in these short-term assays performed in pure buffer, it is important to note that this setup primarily served to assess residual CA activity under conditions resembling those in cyanobacterial culture supernatants. Cyanobacteria are known to secrete proteases during growth [49,50]. To mimic the extracellular environment, the enzymes were incubated with varying concentrations of trypsin, and their residual activity was determined (Fig. S12). In this experiment, the CO_2_ hydration assay was used instead of the esterase assay, since trypsin itself hydrolyzes p-NPA [51]. Under proteolytic stress, the nanogel formulation retained higher activity than the free enzymes, indicating enhanced resistance to proteolytic degradation.

The increased stability of the nanogel compared to the free enzymes can be attributed to several mechanistic effects. The formation of a densely crosslinked polymer network increases overall structural rigidity, thereby reducing conformational flexibility and stabilizing the enzyme structure [52]. As a result, regions of the enzyme that are susceptible to proteolytic attack are less frequently exposed, which decreases the likelihood of degradation [53].

For practical application, however, the long-term performance of CA should be evaluated under cultivation conditions that may include stressors such as native secreted proteases or chelators, which can specifically affect CA stability and activity. As a first step in this direction, we tested the stability of CA in *Synechocystis* culture medium, wherein the cyanobacteria had been grown for up to 6 weeks (Fig. 4D) using the CO_2_ hydration assay. Preliminary tests (Fig. S13) revealed that the buffer capacities of the *Synechocystis* (∼0.2 mM) and *A. platensis* (∼1.8 mM) media were lower than that of the 50 mM Tris-HCl buffer (∼26 mM). To allow for a direct comparison of pH changes, the culture media were adjusted to match the buffer capacity of the Tris-HCl buffer by supplementing with Tris-HCl prior to performing the CO_2_ hydration assay. This ensured consistent conditions across samples, enabling a reliable assessment of CA activity in the presence of aged culture media. As a result, it was possible to measure the activity of both freshly prepared CA variants in growth medium and enzymes present in culture supernatants after 2 and 6 weeks. These time points were chosen to capture the initial physiological response as well as the long-term effects on the cultures. We observed that the activity of freshly prepared CA variants was higher in 50 mM Tris-HCl buffer (Fig. 4C) than in *Synechocystis* medium (Fig. 4D), likely due to the presence of various ions in the medium. Previous studies have shown that salts can inhibit CA activity [10,54]. Activity data from 6-week-old supernatants indicated that all enzyme variants remained active throughout the experiment, with nanogel samples showing the highest activity at the 2-week mark. In summary, the activity measurements provided important initial insights into the long-term stability of the enzymes under culture conditions.

### 3.4 Impact of CA on the growth of *A. platensis*

However, the data above reflect only a single time point in the long-term experiment. To evaluate the effects of different CA formulations on cyanobacterial growth, we first conducted a control experiment to determine whether protein supplementation alone could act as an additional nutrient source. *A. platensis* and *Synechocystis* cultures were grown with or without bovine serum albumin (BSA), but no growth difference was observed (Fig. S14). Based on this, we proceeded to examine the specific impact of CA supplementation on *A. platensis* growth. Based on a previous study where 40 mg L^−1^ culture had been identified as the optimal CA concentration [10], we used this value to calculate the molar concentration of our ST-CA-ST. Accordingly, all experiments were performed with 1.25 μM CA.

**Fig. 5:**
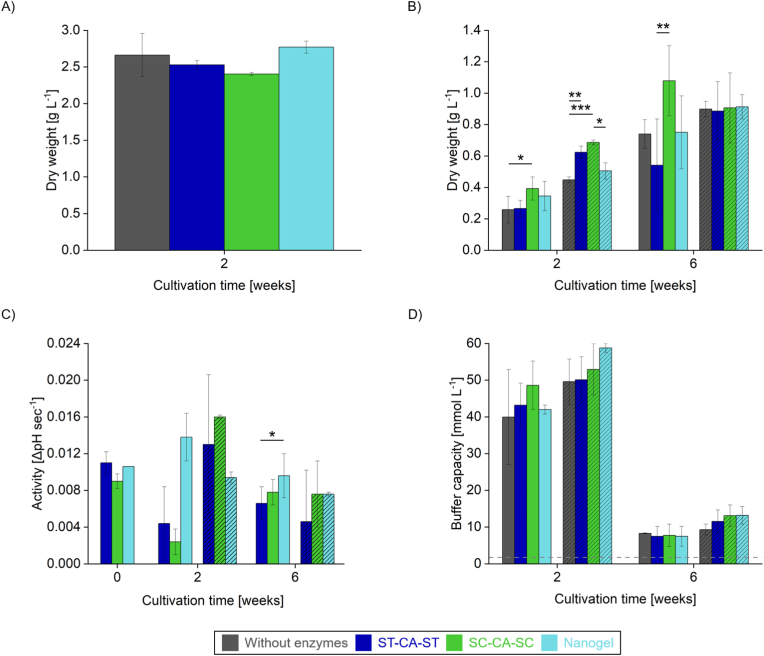
Effect of CA supplementation on the growth of *A. platensis*. Cultures without enzyme supplementation are shown in grey; those with ST-CA-ST in blue, SC-CA-SC in green, and nanogel supplementation in turquoise. (A) Open pond mixed culture was grown and dry weight was determined after 2 weeks. (B) *A. platensis* ecotypes SAG 21.99 (solid bars) or SAG 257.80 (hatched bars) were grown for 6 weeks with biomass determined after 2 and 6 weeks. Initial OD_565_ was adjusted to 0.1. (C) Activity assessment of CA variants in either freshly prepared *A. platensis* media or culture supernatants. Freshly prepared CA was used at 1.25 μM. The same concentration was initially added to the cyanobacteria; residual activity was measured after 2 and 6 weeks. (D) Buffering capacity of the culture supernatants. The dashed line indicates the buffer capacity of the fresh medium. Error bars represent the standard deviation from at least two independent experiments. Statistical significance was assessed via one-way ANOVA followed by the specific post hoc test for multiple comparisons indicated in Table S1 at each tested condition in (B). For (C) only the data points after two and six weeks were used for statistical testing. Comparisons were made among the three CA variants and the cultures without supplementation within each group, and not between different groups. *P*-values are indicated in Table S1. ∗*P* < 0.05, ∗∗*P* < 0.01 and ∗∗∗*P* < 0.001.

We initially investigated a mixed culture of *A. platensis* from an open pond cultivation system, kindly provided by a microalgae farm (ROVAL GmbH, Rockstedt, Germany). The culture is typically dominated by *Arthrospira*, exhibiting both the characteristic helical and straight elongated filamentous forms, alongside various small microorganisms, including ciliates and phytoplankton such as diatoms and *Nannochloropsis* [30]. After two weeks, optical density measurements showed no difference in biomass between cultures with and without CA supplementation (Fig. 5A), likely due to degradation of the CA formulations, possibly caused by the diverse microbial community present in the mixed culture. This degradation was confirmed by SDS-PAGE analysis (Fig. S15A) and corresponding activity measurements. Although different degradation dynamics were detected between the unbound enzymes and the nanogel, no activity was detected in the supernatant after three days of cultivation (Fig. S15B).

To exclude potential interference from non-cyanobacterial microorganisms, pure cultures of the two distinct *A. platensis* ecotypes (see 3.2.) were used instead of the mixed culture. Both ecotypes were cultured for six weeks, with dry biomass measured at weeks 2 and 6 (Fig. 5B). Every two weeks, one-third of the biomass was harvested by centrifugal sedimentation, simulating an industrial process while allowing the CA formulations to remain in the culture for continued activity. The cultivation strategy was designed to reproduce the conditions of unstirred open pond systems, which represent a cost-effective approach for large-scale cyanobacterial production. In such systems, cyanobacterial growth depends on natural sunlight and passive diffusion of nutrients and gases rather than on active mixing or aeration [55]. Although external aeration can enhance cyanobacterial productivity and, in some cases, even outperform CA-based approaches [10], these measures require considerable energy input and negatively affect process economics. Technoeconomic analyses have shown that the power required for mixing and pumping in open raceway ponds constitutes one of the largest operating expenditures, while CO_2_ supply alone has been estimated to account for 40–60 % of total operating costs [56,57]. To reflect these practical constraints, we limited the carbon input to atmospheric air and avoided active mixing, aeration, or artificial illumination, relying instead on CA variants to improve inorganic carbon availability under low-energy conditions.

In this context, the relatively low light intensities measured in our setup (Fig. S1) reflect the seasonal conditions at the study site and the use of a north-facing window, which provides diffuse natural illumination frequently encountered in open pond cultivation systems. In addition, relying solely on atmospheric CO_2_ introduced an inherent carbon limitation, as is typical for unstirred ponds that depend on passive gas exchange rather than active aeration. Together, these conditions create a realistic low-energy environment in which the contribution of CA to inorganic carbon availability becomes particularly relevant.

In the absence of CA supplementation, SAG 257.80 showed better growth than SAG 21.99, likely due to ecotype-specific traits such as differences in photosynthetic efficiency, temperature adaptation, and pigment composition [58]. After the initial two weeks (Fig. 5B, week 2), in SAG 257.80, CA supplementation had a moderate effect, with unbound enzyme variants performing slightly better than the nanogel formulation. In contrast, SAG 21.99 responded more strongly to supplementation, with SC-CA-SC leading to the highest observed growth increase of approximately 50 % during this early cultivation phase, clearly outperforming the other formulations. During photosynthesis, cyanobacteria actively accumulate inorganic carbon through a specialized carbon-concentrating mechanism (CCM) [59]. This process involves the conversion of bicarbonate and CO_2_ within the cells and the concomitant release of hydroxide ions (OH^−^) to the extracellular environment, which leads to an increase in extracellular pH [60] (Fig. S16A). Interestingly, after two weeks, nanogel-treated cultures showed a one-unit drop in pH in comparison to the other conditions, which may have suppressed intrinsic CA production in *A. platensis* [61], and contributed to reduced growth.

However, the growth-promoting effect of CA gradually diminished over time. This decline was more pronounced in SAG 21.99 than in SAG 257.80. By the end of the six-week period, most differences between culture conditions had disappeared, except in SAG 21.99 supplemented with SC-CA-SC, which continued to show a modest improvement. Previous research on *Chlorococcum littorale* demonstrated that elevated CO_2_ concentrations increase intracellular CA activity, leading to acidification that ultimately inhibits growth [62]. A similar mechanism may have occurred in *A. platensis*, where the prolonged activity of the supplemented CA over six weeks could have counteracted the initial growth enhancement. To assess the persistence of CA activity and gain further insight into the underlying processes, *A. platensis* cultures were centrifuged and the cell-free supernatants were analyzed using the CO_2_ hydration assay (Fig. 5C). The activity of freshly added enzymes in untreated *Arthrospira* medium was comparable to that observed in *Synechocystis* medium (Fig. 4D). Since the native CA in *A. platensis* is localized intracellularly [63], any activity detected in the supernatant reflects the presence of the supplemented enzyme. Notably, measurable CA activity persisted for up to six weeks, even though distinct CA bands were no longer visible in SDS-PAGE (Fig. S17). These findings support the hypothesis that the endogenous CA of *A. platensis* already provides near-optimal growth conditions [64,65], thereby limiting the potential benefit of additional CA supplementation.

Further insight into the cultures’ metabolic dynamics was provided by changes in buffer capacity (Fig. 5D). This peaked at week 2 and declined by week 6. Interestingly, all 2-week-old *A. platensis* cultures exhibited buffer capacities above 35 mM, surpassing that of 50 mM Tris-HCl buffer (26 mM, see Fig. S13). The later decrease may be due to increased bicarbonate consumption driven by cell growth or greater production of acidic metabolites, both contributing to the reduction observed by the final time point.

### 3.5 Impact of CA on the growth of *Synechocystis*

**Fig. 6:**
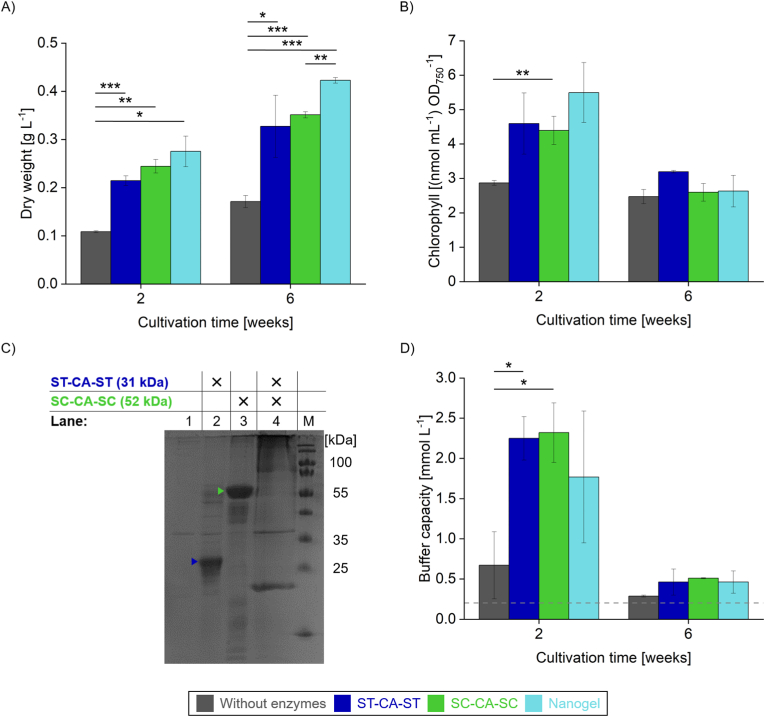
Effect of CA on the growth of *Synechocystis* sp. PCC 6803. Cultures of *Synechocystis* sp. PCC 6803 were grown without enzyme supplementation (grey), with ST-CA-ST (blue), with SC-CA-SC (green) or with nanogel supplementation (turquoise). (A) Cultures were grown for 6 weeks with biomass determined after 2 and 6 weeks. Initial OD_730_ was adjusted to 0.1. (B) Chlorophyll content normalized to OD_750_. (C) SDS-PAGE analysis of culture supernatants after 6 weeks of growth. Lane 1: no enzyme supplementation; lane 2: ST-CA-ST; lane 3: SC-CA-SC; lane 4: nanogel. (D) Buffering capacity of the culture supernatants. The dashed line indicates the buffer capacity of the fresh medium. Error bars in (A), (B) and (D) represent the standard deviation from at least two independent experiments. Statistical significance was assessed via one-way ANOVA followed by the specific post hoc test for multiple comparisons indicated in Table S1 at each tested condition in (A), (B) and (C) (without buffer capacities after six weeks). Comparisons were made among the three CA variants and the cultures without supplementation within each group, and not between different groups. *P*-values are indicated in Table S1. ∗*P* < 0.05, ∗∗*P* < 0.01 and ∗∗∗*P* < 0.001.

Building on the transient effects observed in the SAG strain studies, we conducted further growth experiments with *Synechocystis* to investigate potential strain-specific responses to the CA materials (Fig. 6). Using the same experimental approach as with *A. platensis*, we observed pronounced effects in *Synechocystis* cultures. Notably, nanogel-supplemented cultures consistently reached the highest biomass levels (Fig. 6A), with dry weight closely correlating with the elevated enzymatic activity of the nanogels observed at the 2-week mark (Fig. 4D). Even after six weeks, nanogel-treated cultures showed a 147 % increase in dry biomass compared to those without enzyme supplementation. Given that *Synechocystis* is known to secrete various proteases during the later stages of growth [49,50], the sustained effectiveness of the nanogel formulation likely stems from its enhanced resistance to enzymatic degradation. This highlights its potential for long-term application in cyanobacterial cultures.

To further explore the cultivation process, we quantified chlorophyll content in *Synechocystis* using a spectrophotometric method that normalizes chlorophyll levels to cell density (OD_750_) [29]. Cultures supplemented with CA showed higher chlorophyll production during the first two weeks compared to untreated controls (Fig. 6B). This is particularly relevant given chlorophyll’s growing value as a natural pigment in the food industry, offering antioxidant benefits and a sustainable alternative to synthetic dyes [66].

The increased chlorophyll content likely reflects enhanced CO_2_ availability, which promotes pigment synthesis to support photosynthesis under elevated CO_2_ conditions [67]. However, this effect diminished over time, with chlorophyll levels in CA-treated cultures gradually approaching those of the control. This decline may result from sustained enzymatic activity combined with rising biomass, leading to reduced bicarbonate availability per cell and a possible downregulation of chlorophyll synthesis as a means of energy conservation. This interpretation is supported by the observation of a temporary pH increase observed after two weeks of nanogel treatment (Fig. S16B), a condition known to promote chlorophyll accumulation [68]. By week six, this pH effect had disappeared, aligning with the decline in relative chlorophyll content. The stability of CA during extended cultivation was confirmed by SDS-PAGE analysis of the supernatant from *Synechocystis* cultures (Fig. 6C). Clear single bands were observed for ST-CA-ST and SC-CA-SC, while the nanogels appeared as high-molecular-weight bands, with some remaining in the stacking gel pocket due to their size. This clearly indicated the integrity of the CA materials (see also Fig. S18).

To further understand the effects of CA supplementation on the culture environment, we examined changes in buffer capacity during *Synechocystis* cultivation. Since cyanobacterial growth is known to increase medium buffer capacity [69], we investigated whether this effect is influenced by the presence of CA. Compared to fresh medium, all culture supernatants showed elevated buffer capacities (Fig. 6D), likely due to the secretion of exopolysaccharides, especially during later stages of growth [70]. After two weeks, cultures supplemented with CA exhibited higher buffer capacities than those without enzyme addition. Given that CA activity, and therefore bicarbonate production, remained relatively constant throughout the experiment (Fig. 4D), the initially low biomass likely resulted in limited bicarbonate uptake during early growth. As biomass increased, more bicarbonate was consumed by the growing cells, leading to a gradual decrease in buffer capacity by week six. An additional factor contributing to pH decline may be the enhanced production of acidic metabolites, such as fatty or organic acids, in CA-supplemented cultures [71,72], which could have depleted the accumulated buffer capacity over time.

### 3.6 Practical applicability and reusability of CA nanogels for enhanced cyanobacterial growth

Overall, the results demonstrate that CA supplementation exerts a stronger effect in *Synechocystis* than in *A. platensis*. A key advantage of the nanogel approach over strategies such as surface display is its straightforward transferability: the nanogels can be directly added to diverse photosynthetic microorganisms without requiring genetic modification.

However, as observed for *A. platensis*, the intrinsic CA activity of the host organism must be considered. The weaker growth promotion in *A. platensis* compared to *Synechocystis* is likely attributable to its naturally high endogenous CA activity, which supports rapid growth and thereby diminishes the benefit of additional external CA [[63], [64], [65],^73^]. Consequently, the presented system appears particularly effective in more slowly growing cyanobacteria, where the relative contribution of exogenous CA activity becomes more significant.

This approach may be especially advantageous for large-scale applications such as unstirred open pond reactors, where the simple addition of CA nanogels could enhance carbon availability and overall productivity. From an economic perspective, enzyme reusability and dosage are also important factors. To assess whether comparable biomass yields could be achieved with reduced enzyme levels, all cyanobacterial strains were cultivated for two weeks with either 10 % or 0.1 % of the initial CA concentration (Fig. S19). In all cases, higher CA concentrations resulted in increased biomass formation, consistent with the greater number of catalytic sites available for CO_2_ hydration.

Furthermore, to enable enzyme recovery and reuse, CA nanogels were immobilized on commercially available epoxy-functionalized magnetic beads. Purified SC was covalently attached to the bead surface via its N-terminus and lysine residues, followed by incubation with either ST-CA-ST alone or pre-assembled CA nanogels. Remarkably, catalytic activity was approximately tenfold higher when nanogels were used, and this enhanced activity was maintained over seven consecutive reaction cycles (Fig. S20).

## 4 Conclusion

This study demonstrates the successful development and application of self-assembling all-enzyme nanogels for enhancing CO_2_ bioavailability in cyanobacterial cultures. Unlike previous strategies that required carrier materials to immobilize carbonic anhydrase (CA), our approach employs the SpyTag/SpyCatcher (ST/SC) system to enable carrier-free self-assembly of CA nanogels through precise covalent cross-linking. All components can be heterologously produced in *E. coli*, as the cross-linking domains are genetically fused to the enzyme, eliminating the complex fabrication procedures associated with carriers such as alginate beads [10] or electrospun fibers [9]. When immobilization on solid supports is desired, the nanogels can bind more efficiently to beads due to multivalent ST/SC interactions, allowing higher enzyme loading per binding site. This simplifies production and provides greater flexibility than approaches based on direct CA overexpression in cyanobacteria, making the method readily transferable to other species.

Using a custom-designed pH-based assay, we demonstrated that the CA nanogels retain high catalytic efficiency despite their densely cross-linked structure. The nanogels remained active in cyanobacterial cultures for several weeks, indicating strong resistance to proteolytic degradation and environmental stress, as supported by their sustained activity in the presence of trypsin and EDTA. While CA supplementation had only minor effects on growth and pH regulation in *A. platensis*, *Synechocystis* cultures showed increased biomass and chlorophyll content compared to untreated controls. Although growth differences among CA variants were not statistically significant, the nanogel consistently yielded the highest biomass formation. Furthermore, the reduced inhibition by acetazolamide relative to the free enzyme suggests that CA is stabilized within the nanogel matrix.

Finally, immobilized nanogels on magnetic beads exhibited catalytic activity approximately an order of magnitude higher than the free enzyme and maintained this performance over multiple reuse cycles. These results highlight the robustness and scalability of the nanogel system. Overall, the self-assembling CA nanogels provide a versatile, genetically encodable platform that combines high catalytic stability with easy adaptability to different host systems. Their capacity to function efficiently under ambient conditions makes them promising candidates for biotechnological applications such as carbon capture, enhancement of photosynthetic biomass production, and integration into multi-enzyme cascade reactions.

## CRediT authorship contribution statement

**Marius Stoeckle:** Writing – original draft, Visualization, Methodology, Investigation, Formal analysis. **Carmen M. Domínguez:** Methodology, Investigation, Formal analysis. **Abbey Hanes:** Methodology, Investigation. **Simone Weigel:** Methodology, Investigation. **Alexei Kiselev:** Methodology, Investigation. **Kersten S. Rabe:** Writing – review & editing, Supervision, Formal analysis, Conceptualization. **Christof M. Niemeyer:** Writing – review & editing, Writing – original draft, Supervision, Project administration, Funding acquisition, Conceptualization.

## Funding

This work was supported through the 10.13039/501100009318Helmholtz Association program “Materials Systems Engineering” under the topic “Adaptive and Bioinstructive Materials Systems” (Funding code: 43.33.11). A.H. acknowledges support from the RISE internship program funded by the 10.13039/100021828German Academic Exchange Service (DAAD).

## Declaration of competing interest

The authors declare that they have no known competing financial interests or personal relationships that could have appeared to influence the work reported in this paper.

## Data Availability

Data will be made available on request.
