# Supplementary material for: A carbonic anhydrase-based nanogel for cyanobacterial growth enhancement
Source: Mater Today Bio. 2025 Dec 1;35:102622. doi: 10.1016/j.mtbio.2025.102622 (PMC12731297; doi:10.1016/j.mtbio.2025.102622)
Supplement: Multimedia component 1 [file mmc1.docx]

Supporting information

**A Carbonic Anhydrase-Based Nanogel for Cyanobacterial Growth Enhancement**

Marius Stoeckle, Carmen M. Domínguez, Abbey Hanes, Simone Weigel, Alexei Kiselev, Kersten S. Rabe and Christof M. Niemeyer

**Table of contents**

**Supplementary figures**

Figure S1: Cultivation setup of the cyanobacterial strains

Figure S2: SDS-PAGE gels of the CA purification

Figure S3: Histogram of Feret diameter as determined by image processing of the atomic force microscopy images using ImageJ

Figure S4: Electron microscopic images of CA nanogels

Figure S5: Microscopy images of the *A. platensis* ecotypes used in this study

Figure S6: Representative fluorescence microscopy images illustrating nanogel localization in *A. platensis* SAG 21.99

Figure S7: Representative fluorescence microscopy images illustrating nanogel localization in *A. platensis* SAG 257.80

Figure S8: Representative fluorescence microscopy images illustrating nanogel localization in *Synechocystis* sp. PCC 6803

Figure S9: Fluorescence microscopy image illustrating nanogel localization in *A. platensis* SAG 257.80 after two hours of incubation

Figure S10: Activity determination of CA variants using *p*-nitrophenyl acetate (p-NPA)

Figure S11: Biochemical characterization of CA variants under different conditions

Figure S12: Protease treatment of CA variants

Figure S13: Buffer capacity and pH changes of used media

Figure S14: Influence of bovine serum albumin (BSA) on the growth of the *A. platensis* ecotypes and *Synechocystis* sp. PCC 6803

Figure S15: Determination of CA stability and activity in mixed cultures derived from an open pond cultivation system

Figure S16: pH values of the culture supernatants

Figure S17: SDS-PAGE gel of the *A. platensis* culture supernatant

Figure S18: Uncropped SDS-PAGE gel of the *Synechocystis* sp. PCC 6803 culture supernatant after 6 weeks of growth

Figure S19: Influence of different CA concentrations on the growth of the *A. platensis* ecotypes and *Synechocystis* sp. PCC 6803

Figure S20: Catalytic cycles of immobilized ST-CA-ST and CA nanogels on magnetic beads

**Supplementary tables**

Table S1: Overview of post hoc tests used in this study and the resulting *P*-values

**References**

**Supplementary figures**

**
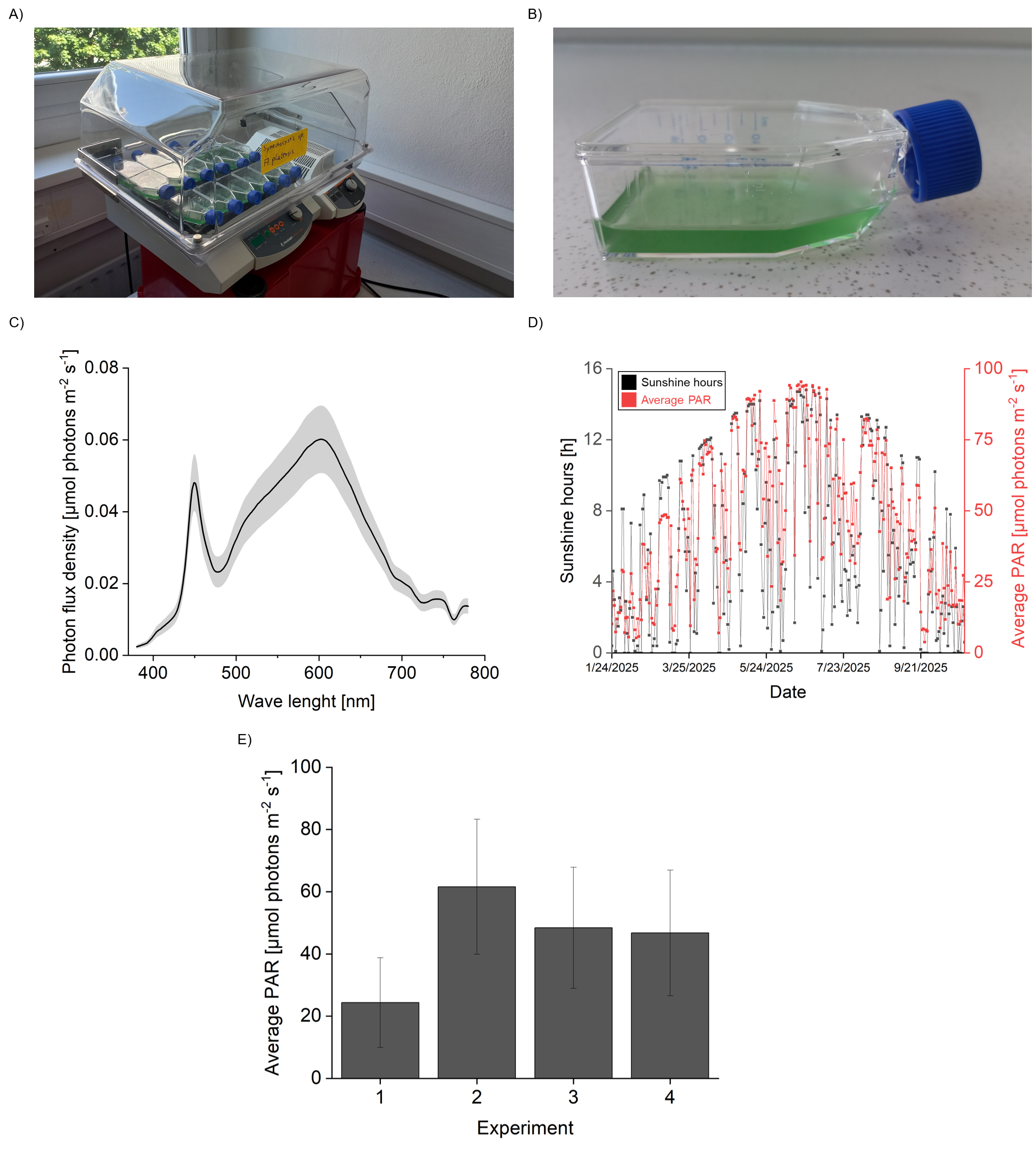
**

Fig. S1. Cultivation setup of the cyanobacterial strains designed to mimic the conditions of an unstirred open pond system. (A) Incubator operated under atmospheric CO_2_ levels at 25 °C. No shaking or forced aeration was applied, reproducing the low-energy conditions of open pond cultivation. The incubator was placed 50 cm from a north-facing window, which served as the sole light source and provided a natural day–night cycle. The corresponding hours of sunlight are shown in panel (D). All cultures within each experiment were incubated simultaneously in the same growth chamber, ensuring that all samples experienced identical environmental conditions. (B) Culture tube equipped with a cap containing a filter membrane that allowed passive gas exchange with the surrounding air while preventing contamination. This setup ensured a stable, diffusion-driven CO_2_ supply and reflected the minimal technical intervention typical of open pond systems. (C) Exemplary spectral properties of the light inside the incubator. Spectra were recorded using a LI-180 spectrometer (LI-COR) across several experiments, allowing calculation of the standard deviation. Wavelengths from 380–780 nm were measured. (D) Sunshine hours and average photosynthetically active radiation (PAR). Sunshine hours were obtained from a nearby German Weather Service station (https://www.dwd.de/; data from Rheinstetten), and irradiance was measured at a local weather station operated by the Institute for Meteorology and Climate Research (KIT). Irradiance values (W m^-2^) were recorded at 10-min intervals over 24 h, and daily mean irradiance was calculated. Using a published conversion [1] (1 W m^-2^ ≈ 2.02 µmol photons m^-2^ s^-1^), the mean irradiance was converted into PAR. Based on comparison of outdoor measurements with photon flux densities recorded inside the incubator, a maximum of 13% of the outdoor photon flux density reached the cultivation chamber. Accordingly, the converted outdoor PAR values were multiplied by 0.13 to estimate the effective PAR inside the incubator. (E) Exemplary PAR values for the four six-week cyanobacterial growth experiments. Mean PAR values were calculated using the data in panel (D), with standard deviations derived from daily values within each experiment. Experiment 1: January 28 – March 11, 2025; Experiment 2: April 2 – May 14, 2025; Experiment 3: August 12 – September 23, 2025; Experiment 4: August 13 – September 24, 2025.

**
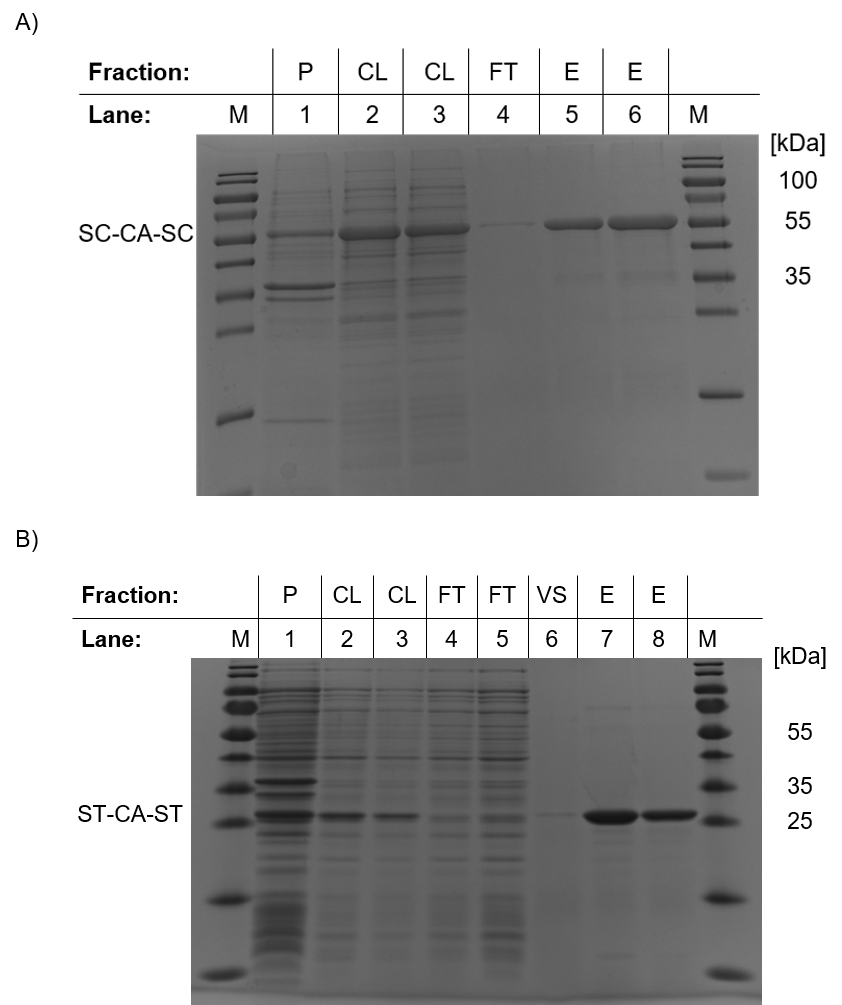
**

Fig. S2. SDS-PAGE gels of the CA purification. P = pellet fraction; CL = clear lysate; FT = fraction, that did not bind to the Ni-NTA column; VS = fraction that flowed through the VivaSpin concentrator; E = obtained protein after concentration. (A) Purification of SC-CA-SC. (B) Purification of ST-CA-ST.


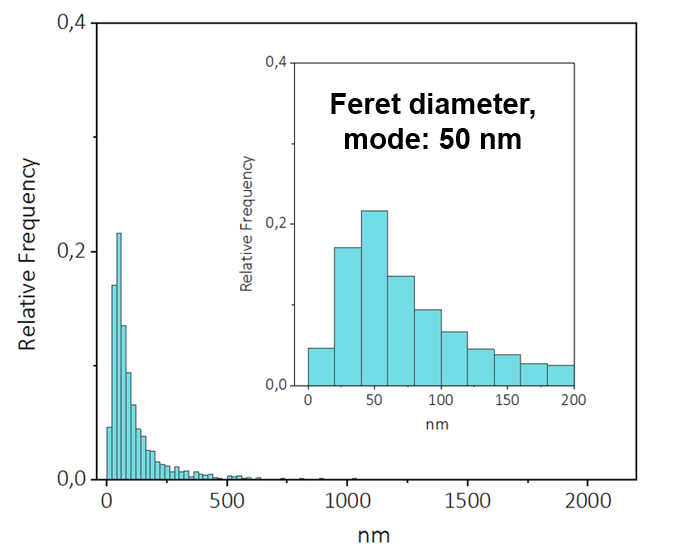


Fig. S3. Histogram of Feret diameters obtained from image analysis of atomic force microscopy (AFM) images using ImageJ. The main panel shows the complete particle size distribution, while the inset highlights particles with Feret diameters up to 200 nm. The histogram was generated with a bin width of 20 nm. Due to the strongly right-skewed distribution, the mode value was used to represent the most frequent particle size.


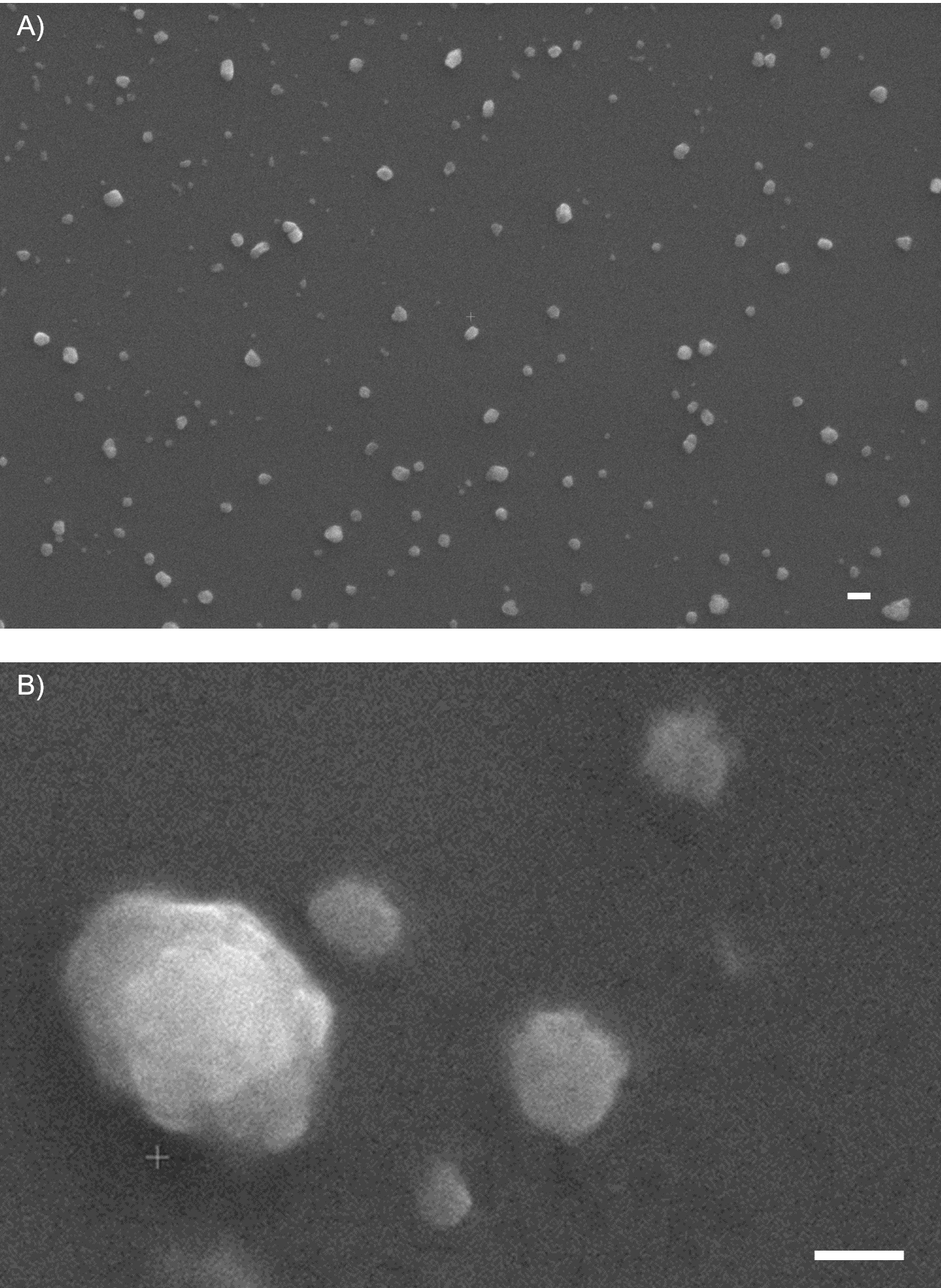


Fig. S4. Electron microscopic images of CA nanogels. (A) Overview of nanogels. (B) Enlarged view of the nanogel morphology. Scale bar: 100 nm. Particle dimensions agree well with AFM and DLS data, although larger particles were sometimes observed (Fig. S4B), likely due to drying-induced aggregation during sample preparation, similar to monolithic AEH formation (Fig. 1C).


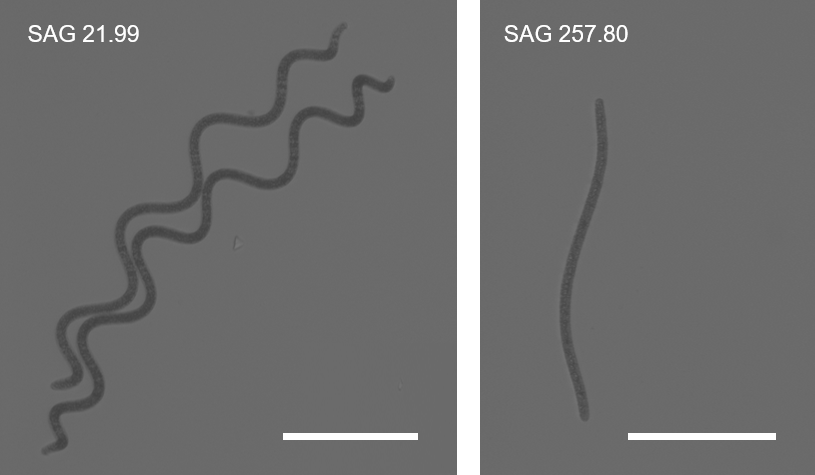


Fig. S5. Microscopy images of the *A. platensis* ecotypes used in this study. Scale bar: 100 µm.


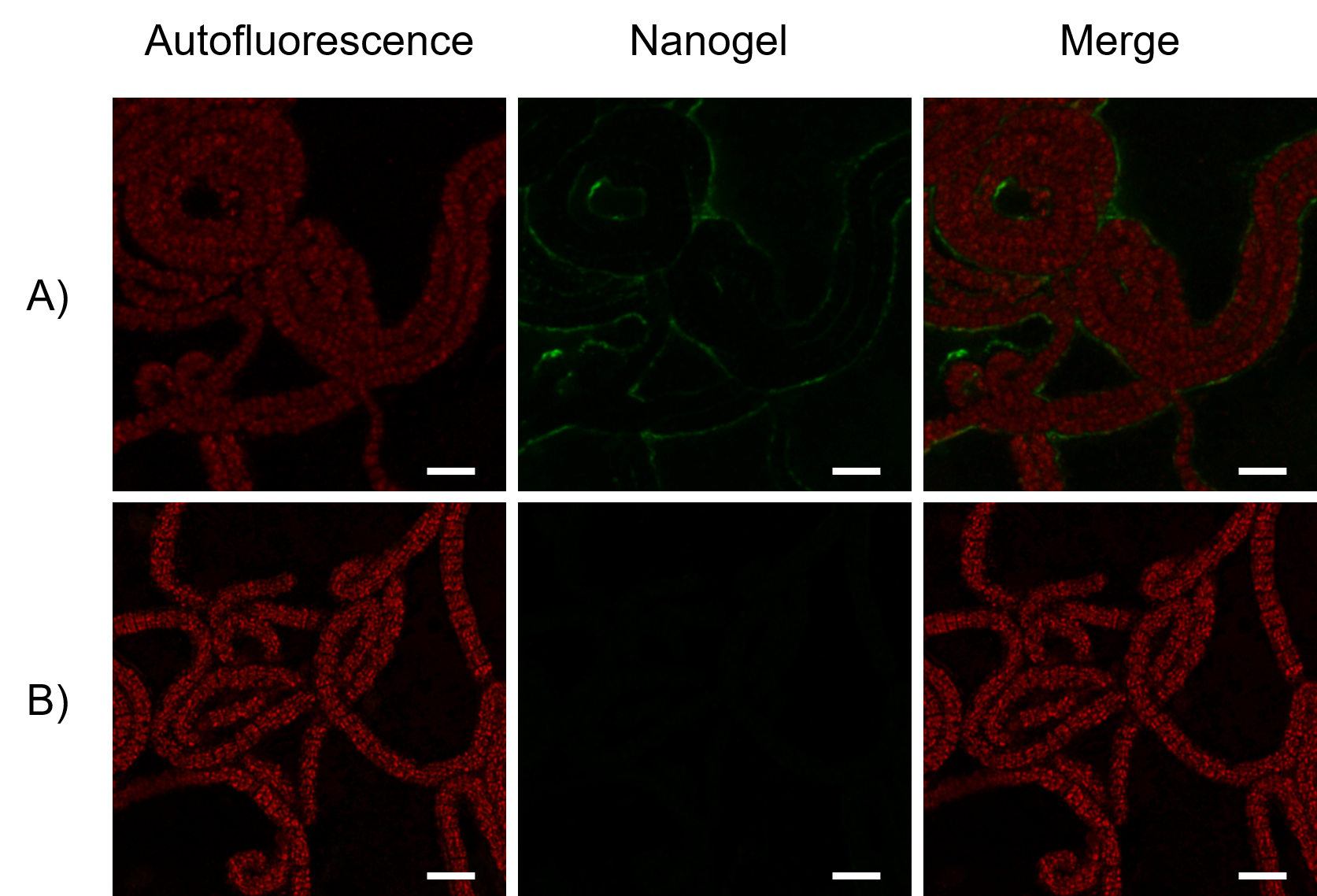


Fig. S6. Representative fluorescence microscopy images illustrating nanogel localization in *A. platensis* SAG 21.99. Cultures were grown for two weeks in the presence of CA nanogels, and cells were visualized by their autofluorescence (red, excitation 561 nm, emission 579 nm). (A) Cultures incubated with nanogels tagged with ST-eGFP-ST (green, excitation 488 nm, emission 516 nm). (B) Cultures incubated with nanogels lacking eGFP. Images in (A) and (B) were acquired and exported using identical settings. Scale bar: 10 µm.


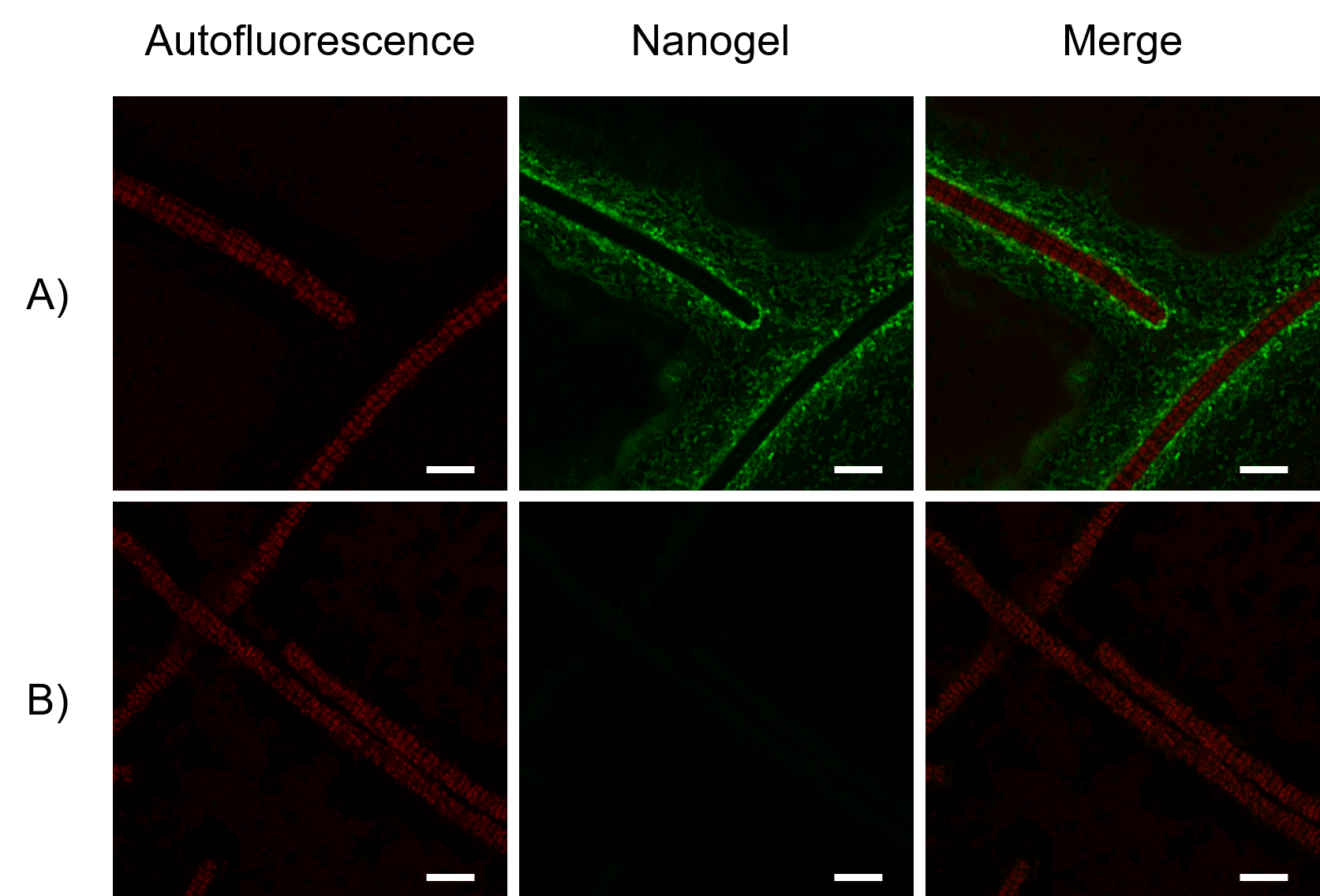


Fig. S7. Representative fluorescence microscopy images illustrating nanogel localization in *A. platensis* SAG 257.80. Cultures were grown for two weeks in the presence of CA nanogels, and cells were visualized by their autofluorescence (red, excitation 561 nm, emission 579 nm). (A) Cultures incubated with nanogels tagged with ST-eGFP-ST (green, excitation 488 nm, emission 516 nm). (B) Cultures incubated with nanogels lacking eGFP. Images in (A) and (B) were acquired and exported using identical settings. Scale bar: 10 µm.


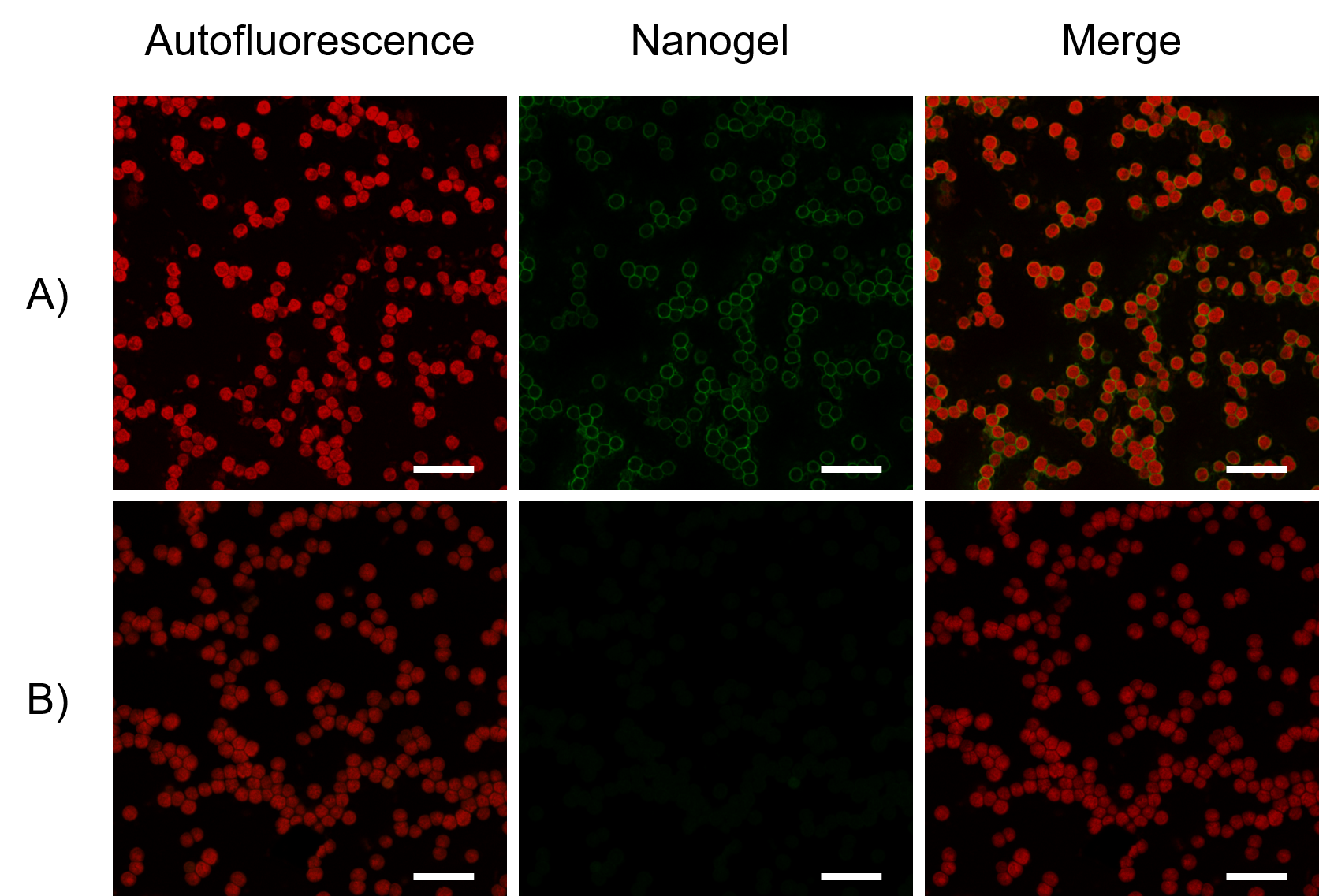


Fig. S8. Representative fluorescence microscopy images illustrating nanogel localization in *Synechocystis* sp. PCC 6803. Cultures were grown for two weeks in the presence of CA nanogels, and cells were visualized by their autofluorescence (red, excitation 561 nm, emission 579 nm). (A) Cultures incubated with nanogels tagged with ST-eGFP-ST (green, excitation 488 nm, emission 516 nm). (B) Cultures incubated with nanogels lacking eGFP. Images in (A) and (B) were acquired and exported using identical settings. Scale bar: 10 µm.


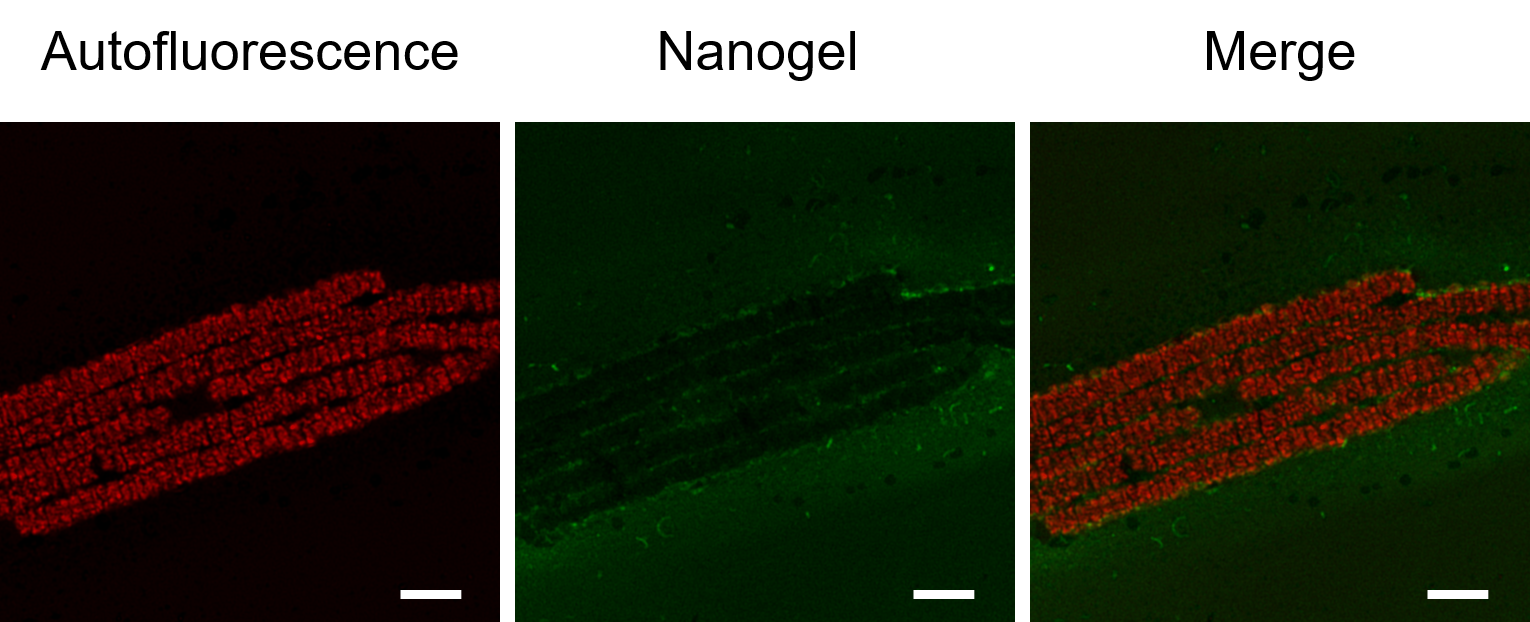


Fig. S9. Representative fluorescence microscopy image illustrating nanogel localization in *A. platensis* SAG 257.80 after two hours of incubation. Cultures were grown for two hours in the presence of CA nanogels, and cells were visualized by their autofluorescence (red, excitation 561 nm, emission 579 nm). Nanogels were tagged with ST-eGFP-ST (green, excitation 488 nm, emission 516 nm). The image was acquired and exported using identical settings as in Fig. 3F. Scale bar: 10 µm.


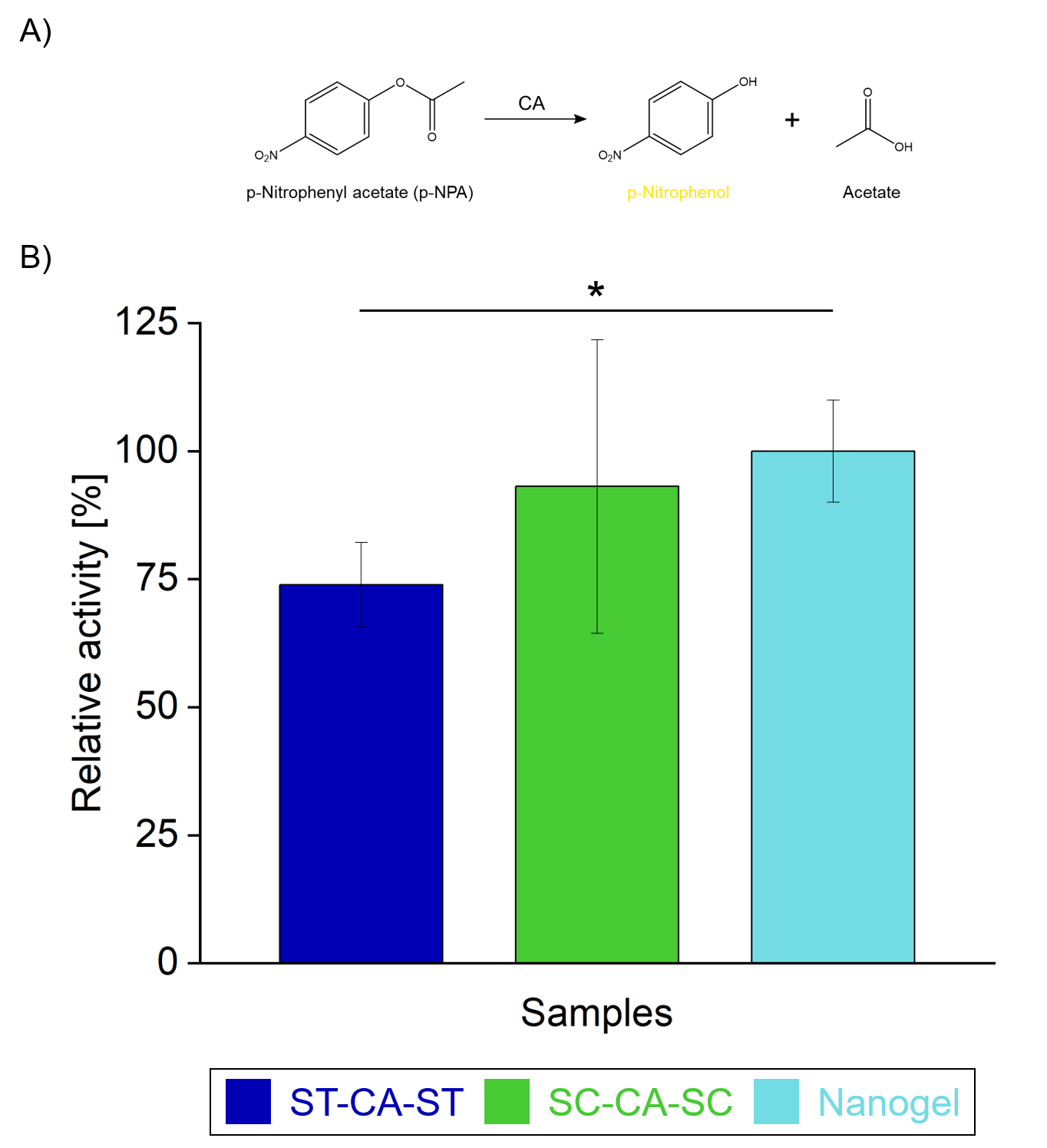


Fig. S10. Activity determination of CA variants using *p*-nitrophenyl acetate (p-NPA). **(**A) Reaction scheme of the esterase assay. *p*-Nitrophenol formation was detected spectrophotometrically at the characteristic absorbance at 348 nm. (B) Enzymatic activity comparison of the CA variants (ST-CA-ST in blue, SC-CA-SC in green) at 30 °C. The nanogel (turquoise) was set to 100%. All reactions contained the same number of CA monomers (i.e., 4 µM active sites). The error bars represent the standard deviation from two independent experiments. Statistical significance was assessed via one-way ANOVA followed by the specific post hoc test for multiple comparisons indicated in Table S1. *P*-values are indicated in Table S1. **P* < 0.05.


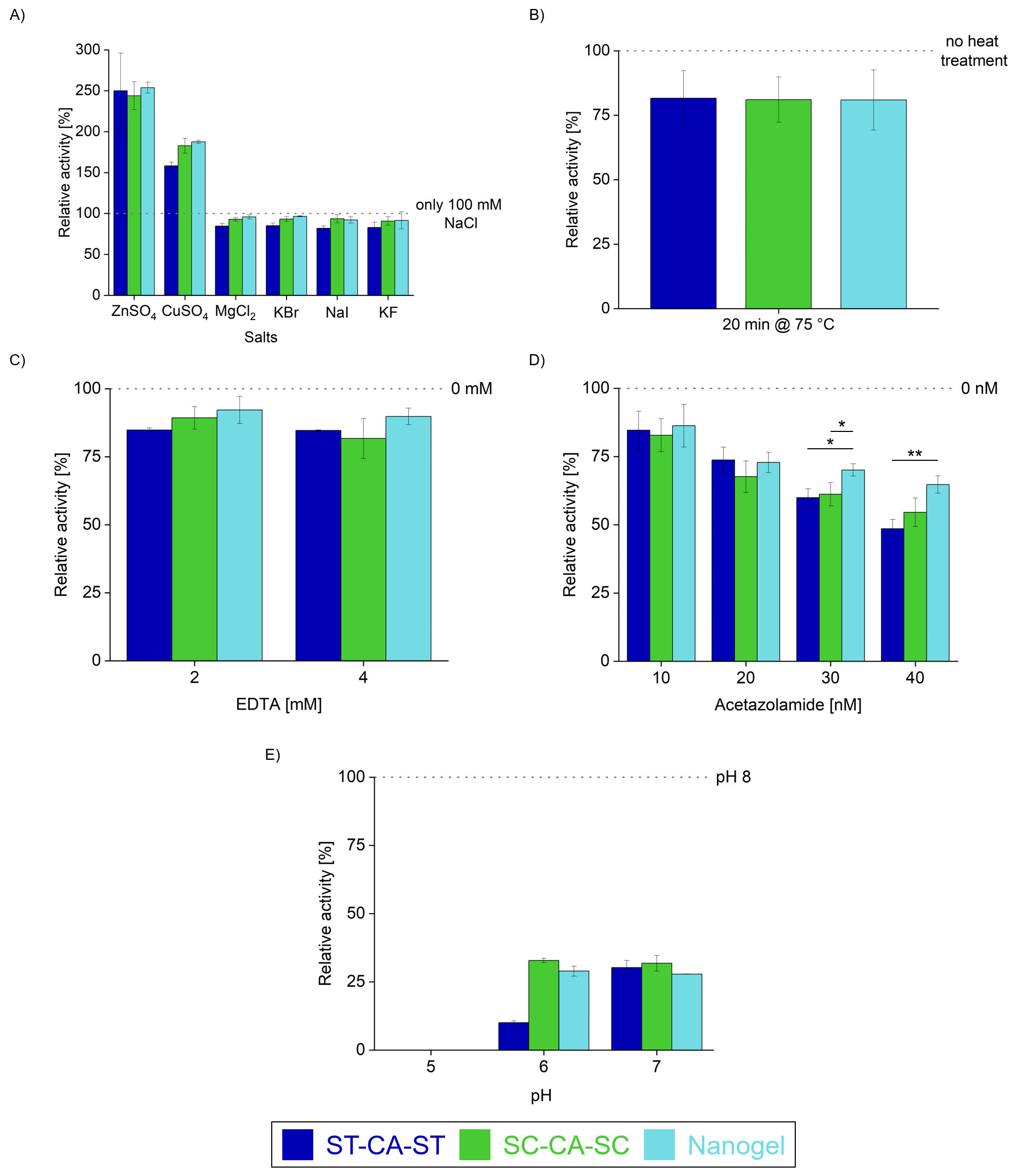


Fig. S11. Biochemical characterization of CA variants under different conditions. Enzymatic activity was determined using p-NPA as substrate and is shown as a relative value, with 100% representing the activity under control conditions (indicated by the dashed line). (A) Influence of 7 mM salt solutions on enzyme activity. (B) Residual activity after heat treatment at 75 °C for 20 min followed by cooling. (C) Effect of the zinc-chelating agent EDTA. (D) Influence of the inhibitor acetazolamide on CA activity. (E) Activity at different pH values measured using the universal Britton–Robinson buffer. Error bars represent the standard deviation from at least two independent experiments. Statistical significance was assessed via one-way ANOVA followed by the specific post hoc test for multiple comparisons indicated in Table S1 at each tested condition (EDTA concentration and acetazolamide concentration). Comparisons were made only among the three CA variants within each group, and not between different groups. *P*-values are indicated in Table S1. **P* < 0.05 and ***P* < 0.01.

**
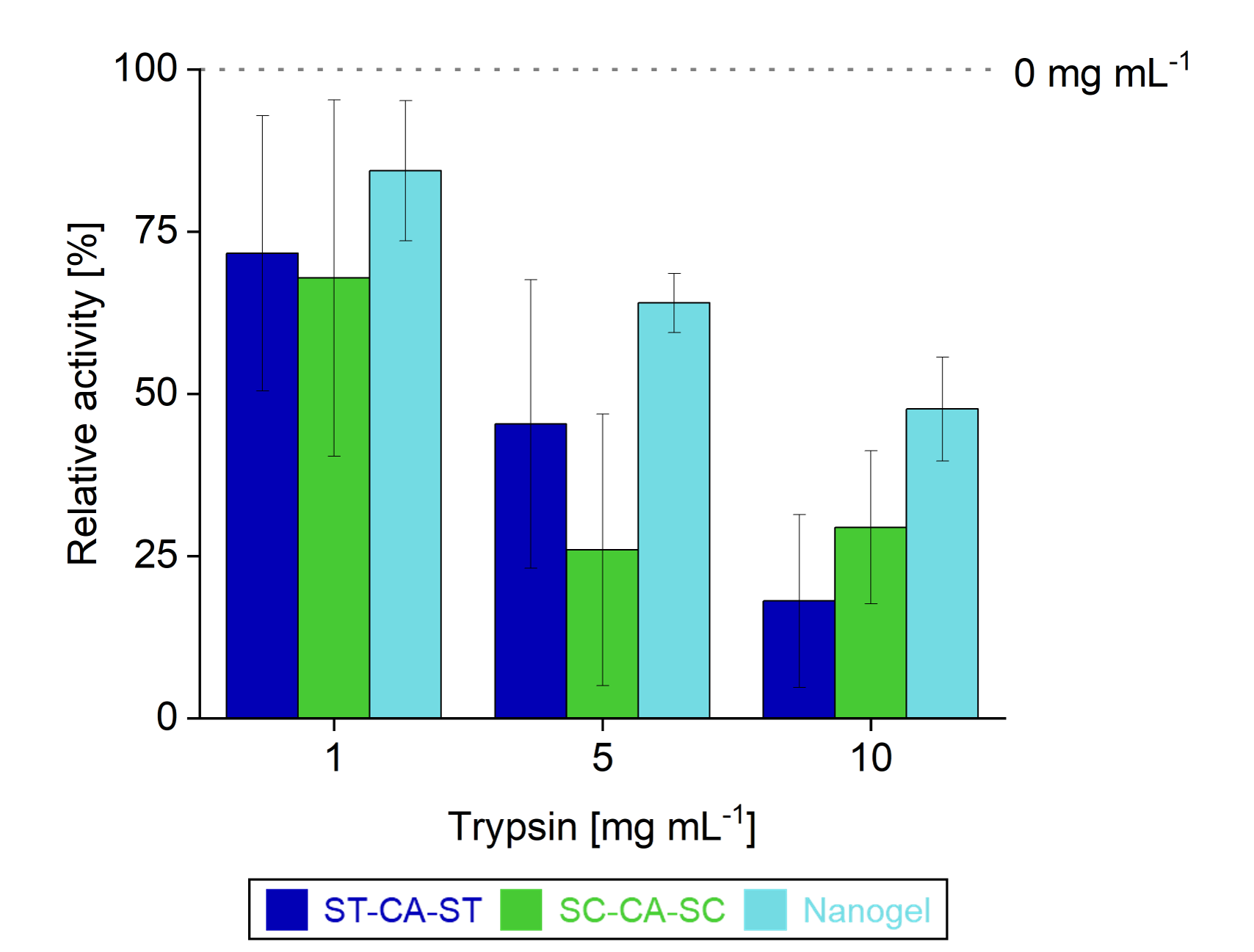
**

Fig. S12. Protease treatment of CA variants. Enzymatic activity was determined using the CO_2_ hydration assay. Error bars represent the standard deviation from at least two independent experiments. Statistical significance was assessed via one-way ANOVA followed by the specific post hoc test for multiple comparisons indicated in Table S1 at two tested conditions (1 mg mL^-1^ and 5 mg mL^-1^). Comparisons were made only among the three CA variants within each group, and not between different groups. *P*-values are indicated in Table S1. No statistical difference was observed.


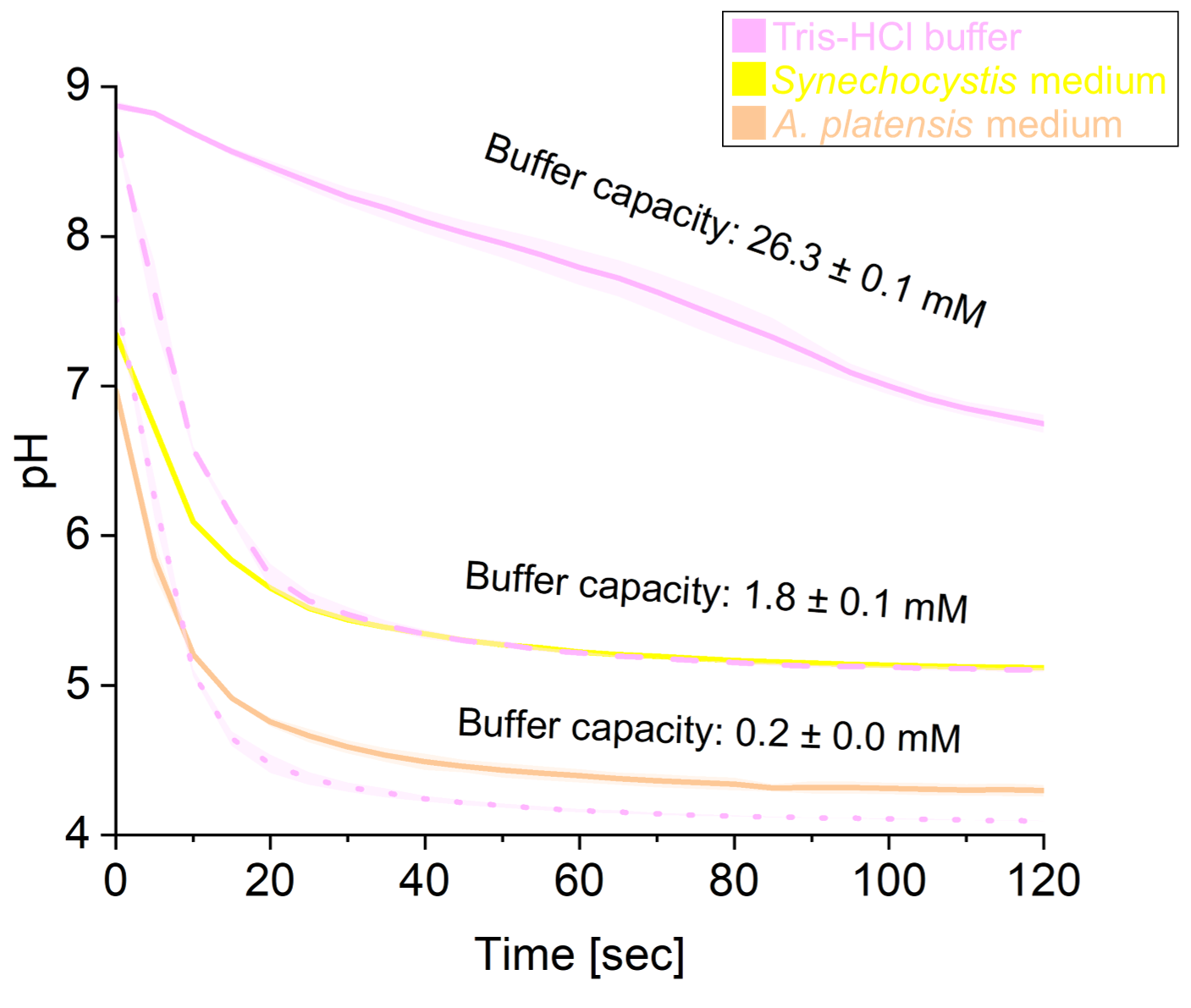


Fig. S13. Buffer capacity and pH changes of used media. pH was monitored while CO₂ was bubbled through 50 mM Tris-HCl buffer (purple), *A. platensis* medium (yellow), or *Synechocystis* medium (orange). Buffer capacities, determined by titration with HCl (see Methods Section), are given on top of the curves. Note that the buffer capacities of the cyanobacterial culture media are significantly lower than that of the 50 mM Tris-HCl buffer. Titrations of Tris-HCl buffer, adjusted to match the buffering capacities of the culture media, result in pH changes shown by the dashed purple lines. To allow for a direct comparison of pH changes, the culture media were supplemented with Tris to match the buffer capacity of the 50 mM Tris-HCl buffer prior to performing the CO_2_ hydration assay. Error bars indicate the standard deviation from two independent experiments.


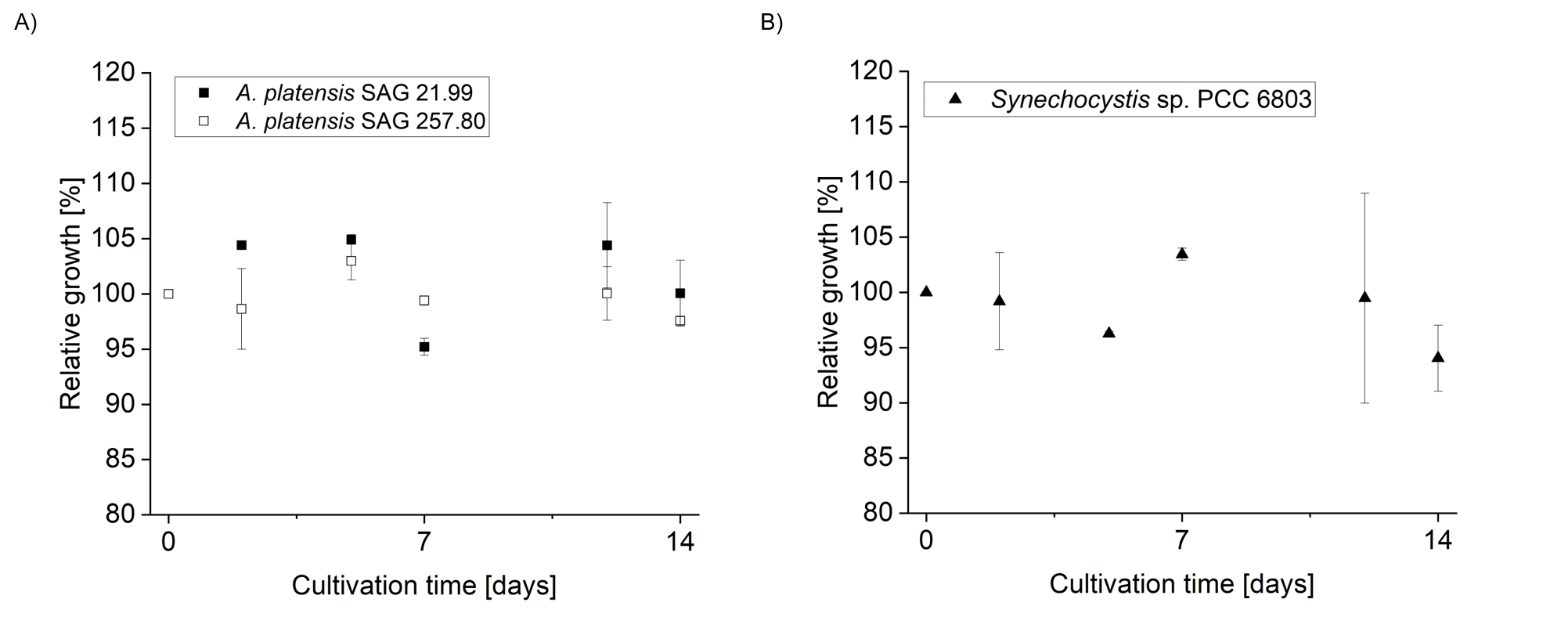


Fig. S14. Influence of bovine serum albumin (BSA) on the growth of the *A. platensis* ecotypes and *Synechocystis* sp. PCC 6803. (A) Cultures of *A. platensis* SAG 21.99 (filled squares) and SAG 257.80 (unfilled squares) and (B) *Synechocystis* sp. PCC 6803 (triangles) were grown with BSA supplementation. Optical densities were recorded at defined time points, and relative growth enhancement was determined by comparing values to unsupplemented control cultures, which were set to 100%. The error bars represent the standard deviation from two independent experiments.


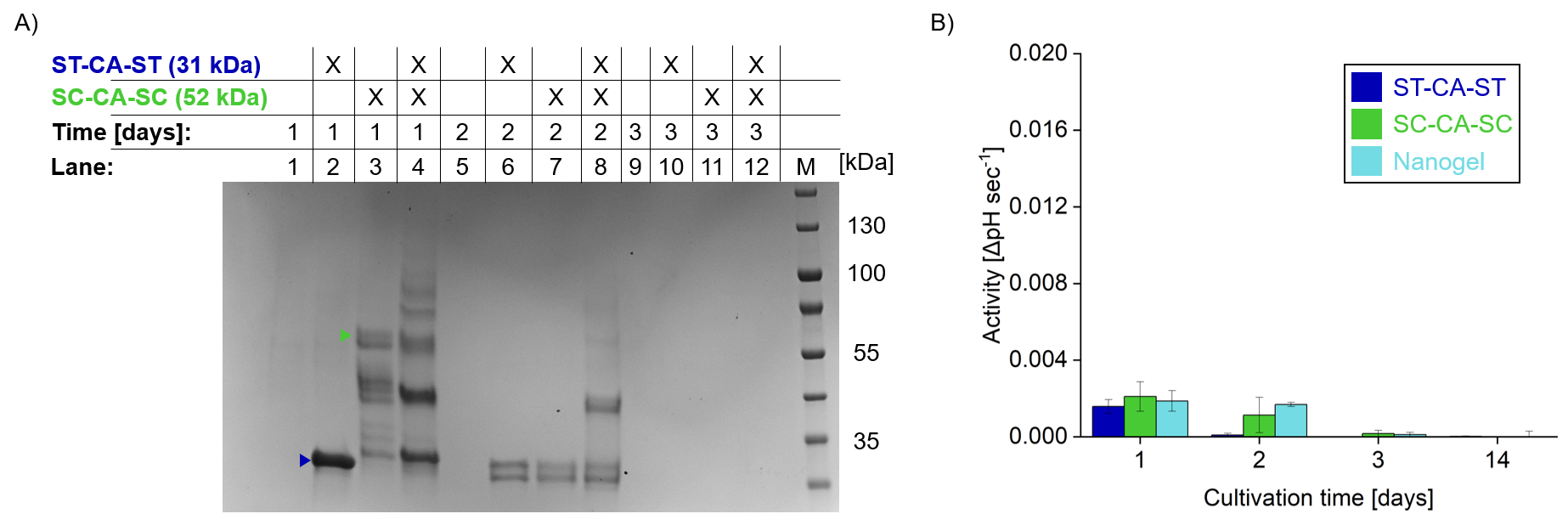


Fig. S15. Determination of CA stability and activity in mixed cultures derived from an open pond cultivation system. Mixed cultures (as described in the main text) were incubated for 1, 2, and 3 days in the presence of free CA or the nanogel formulation. Cell-free supernatants were used for (A) SDS-PAGE analysis after tenfold concentration by ultrafiltration and (B) activity determination using the CO_2_ hydration assay. Error bars represent the standard deviation from two independent experiments.

**
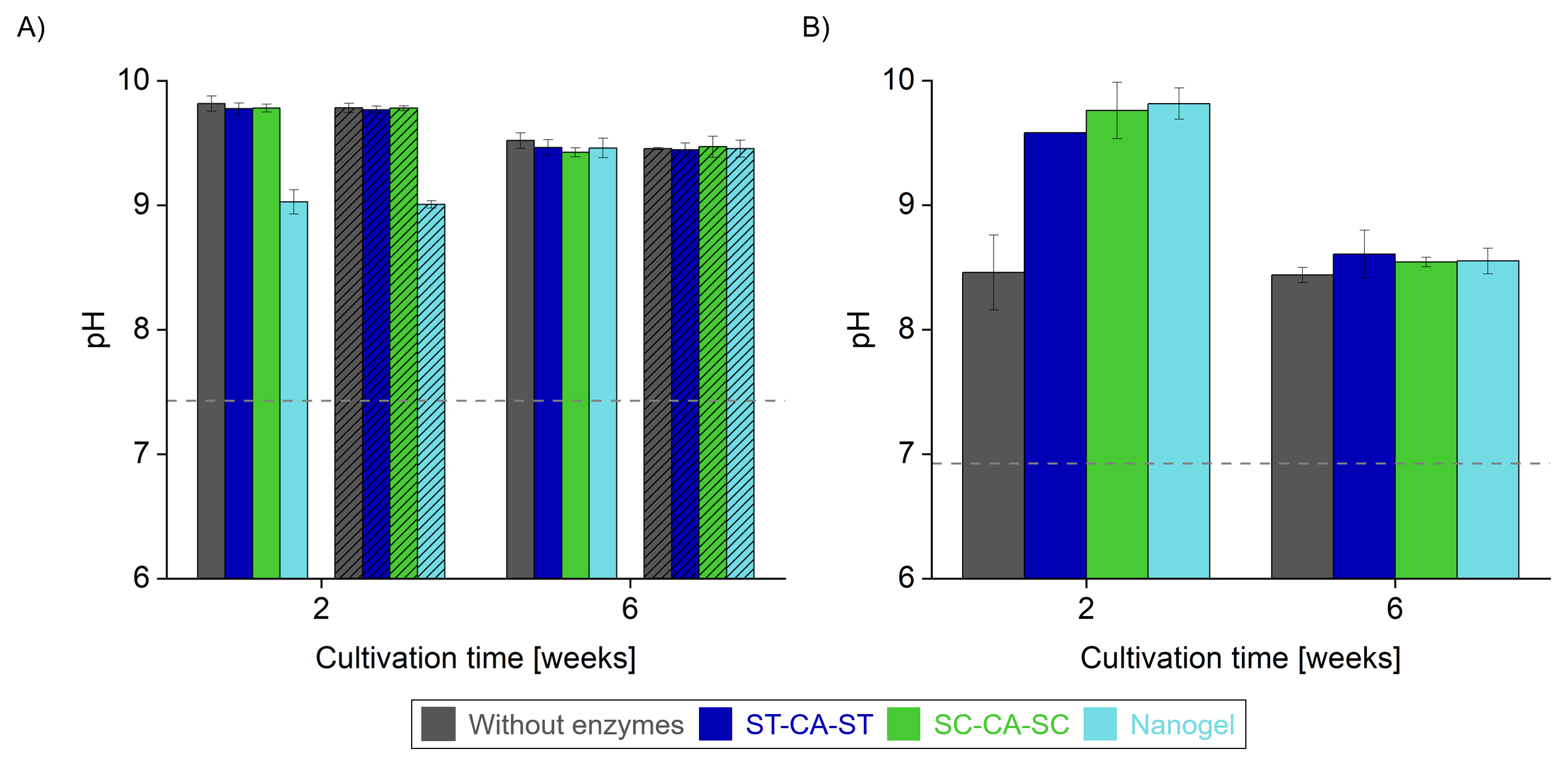
**

Fig. S16. pH values of the culture supernatants. (A) Cultures of *A. platensis* SAG 21.99 (solid bars) and *A. platensis* SAG 257.80 (hatched bars) as well as (B) *Synechocystis* sp. PCC 6803, were centrifuged, and the pH of the resulting cell-free supernatant was measured. Cultures were supplemented with either no enzyme (gray), ST-CA-ST (blue), SC-CA-SC (green) or nanogel (turquoise). The dashed line shows the media’s pH prior to cultivation. The error bars represent the standard deviation from two independent experiments.


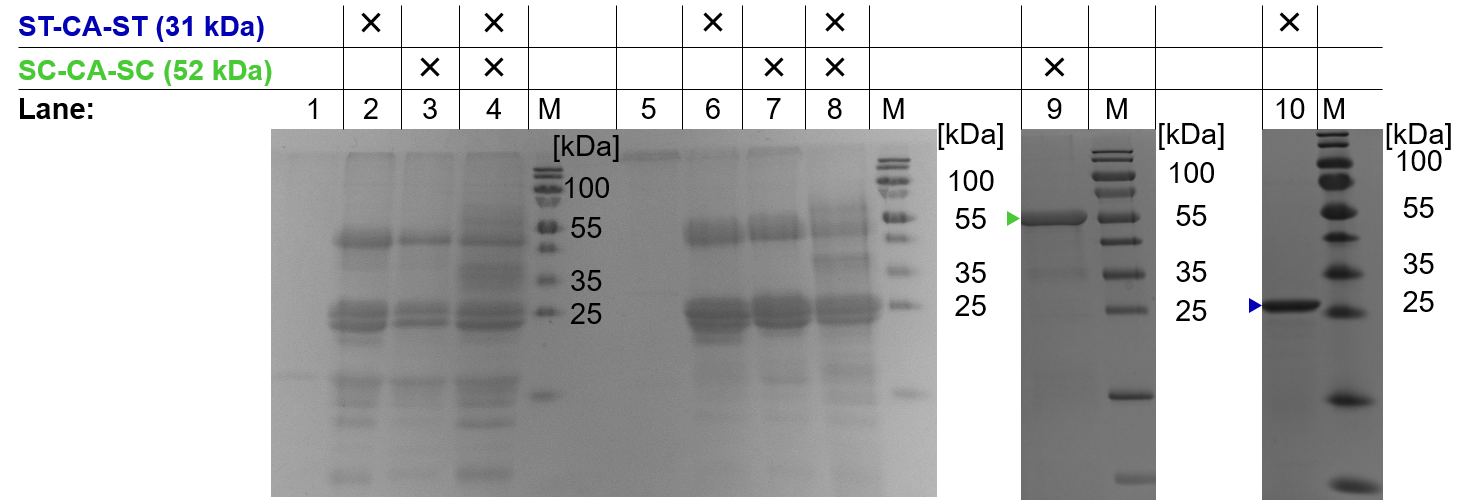


Fig. S17. SDS-PAGE gel of the *A. platensis* culture supernatant. After 6 weeks, the culture supernatant was concentrated and analyzed by SDS-PAGE (lanes 1-4: supernatant of SAG 21.99, lanes 5-8: supernatant of SAG 257.80). Lane 1, 5: no CA supplementation; lane 2, 6: ST-CA-ST; lane 3, 7: SC-CA-SC; lane 4, 8: nanogel. For better size estimation, the lanes containing purified CA samples from Figure S1 are shown in lane 9 (SC-CA-SC) and lane 10 (ST-CA-ST). Note that, unlike the gel showing the supernatant from *Synechocystis sp.* PCC 6803 (Fig. S18), no high-molecular-weight bands corresponding to CA nanogels are visible here (lane 4, 8). However, two additional bands appear at 40 kDa and 65 kDa, which may represent degradation products. A comparison between samples without enzyme addition (lanes 1, 5) and those with added CA suggests that CA supplementation may induce the production of various proteins. These additional bands could be associated with exopolysaccharides, which in *A. platensis* can contain up to 55% protein and whose synthesis is stimulated by elevated bicarbonate levels. This hypothesis is further supported by the fact that exopolysaccharide production typically begins in the later stages of growth [2, 3].


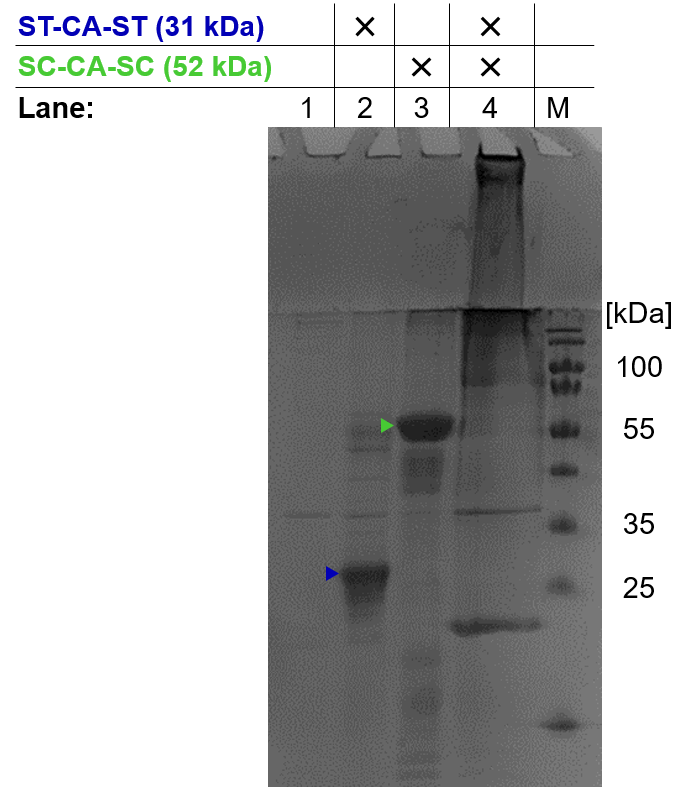


Fig. S18. Uncropped SDS-PAGE gel of the *Synechocystis* sp. PCC 6803 culture supernatant after 6 weeks of growth. Lane 1: no enzyme supplementation; lane 2: ST-CA-ST; lane 3: SC-CA-SC; lane 4: nanogel. Note the presence of high-molecular weight materials inside the stacking gel.


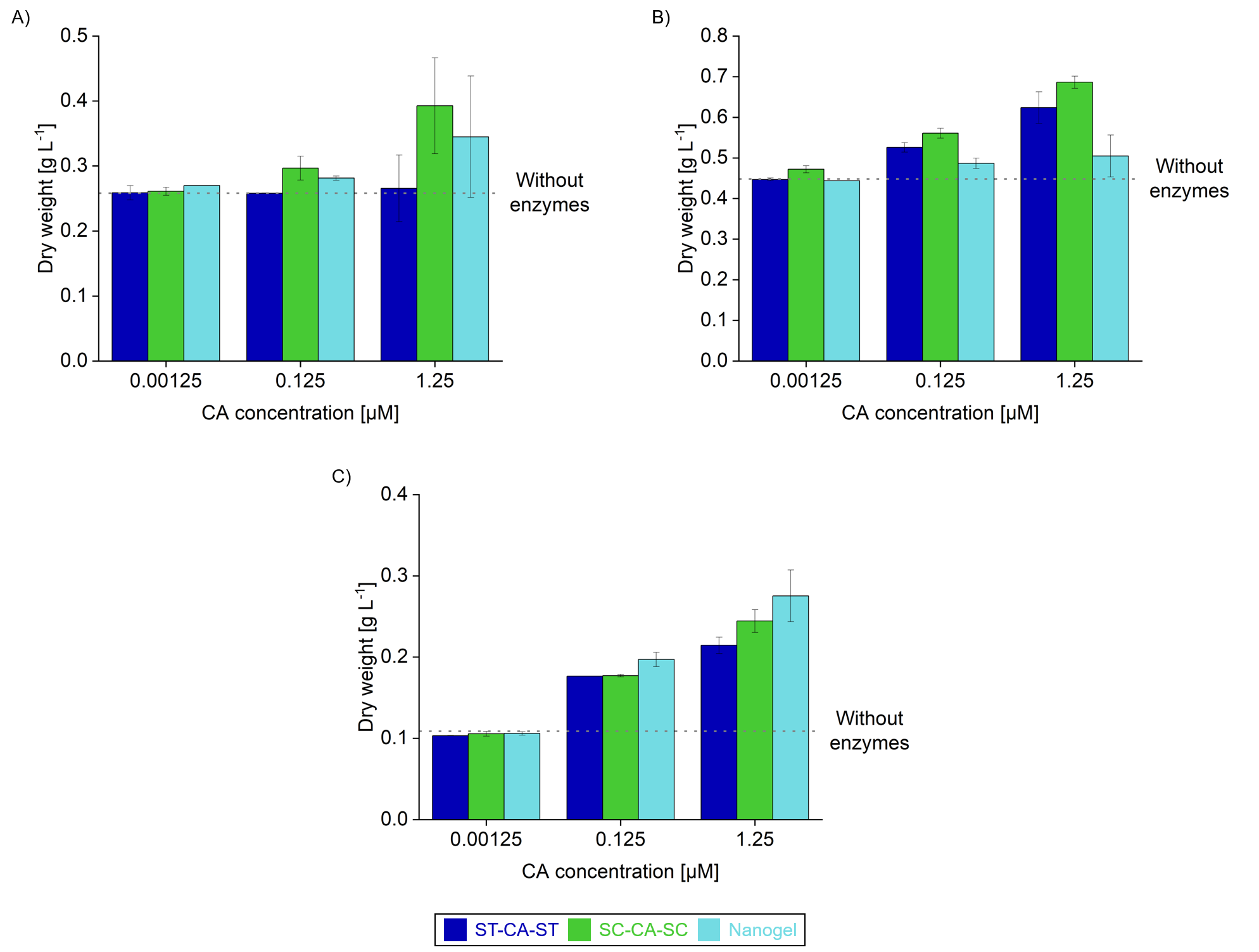


Fig. S19. Influence of different CA concentrations on the growth of the *A. platensis* ecotypes and *Synechocystis* sp. PCC 6803. Cultures of (A) *A. platensis* SAG 21.99, (B) SAG 257.80 and (C) *Synechocystis* sp. PCC 6803 were grown in the presence of CA variants with either 0.125 µM or 1.25 nM. For better comparison, the results of 1.25 µM (main figures) are also shown. Optical densities were recorded after two weeks. The error bars represent the standard deviation from two independent experiments.


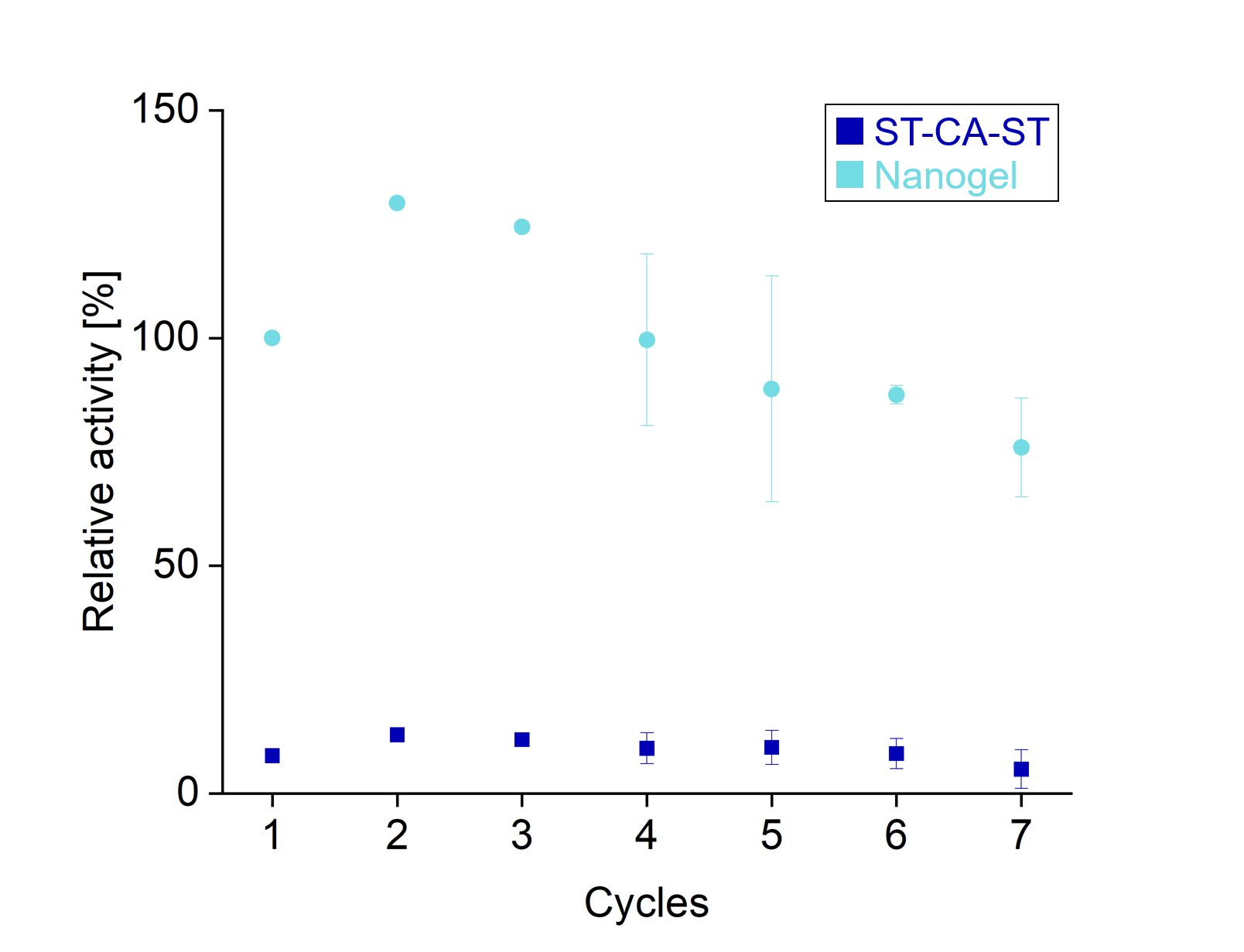


Fig. S20. Catalytic cycles of immobilized ST-CA-ST and CA nanogels on magnetic beads. This experiment was designed to evaluate the reusability and catalytic stability of the immobilized enzyme systems. Magnetic beads were functionalized with SC and subsequently incubated with either ST-CA-ST or pre-assembled CA nanogels. After covalent coupling, the beads were washed three times until no residual enzyme activity was detected in the supernatant. Catalytic activity was determined using p-NPA as the substrate, with the activity remaining after the first wash cycle set to 100%. The beads were then washed and reused in consecutive catalytic cycles. Error bars represent the standard deviation from two independent experiments.

Table S1. Overview of post hoc tests used in this study and the resulting *P*-values. In cases not included, the dataset size was insufficient for statistical evaluation.

| Experiment | Pair comparison | Post hoc test after ANOVA | *P*-value |
| --- | --- | --- | --- |
| Fig. 4C | ST-CA-ST / SC-CA-SC | Tukey HSD | 0.46 |
|  | ST-CA-ST / Nanogel |  | 0.032 |
|  | SC-CA-SC / Nanogel |  | 0.009 |
| Fig. 4D (0 weeks) | ST-CA-ST / SC-CA-SC | Tukey HSD | 0.772 |
|  | ST-CA-ST / Nanogel |  | 0.978 |
|  | SC-CA-SC / Nanogel |  | 0.892 |
| Fig. 4D (2 weeks) | ST-CA-ST / SC-CA-SC | Tukey HSD | 0.972 |
|  | ST-CA-ST / Nanogel |  | 0.487 |
|  | SC-CA-SC / Nanogel |  | 0.415 |
| Fig. 4D (6 weeks) | ST-CA-ST / SC-CA-SC | Tukey HSD | 0.137 |
|  | ST-CA-ST / Nanogel |  | 0.084 |
|  | SC-CA-SC / Nanogel |  | 0.945 |
| Fig. 5B (2 weeks, SAG 21.99) | No enzymes / ST-CA-ST | Tukey HSD | 0.979 |
|  | No enzymes / SC-CA-SC |  | 0.036 |
|  | No enzymes / Nanogel |  | 0.439 |
|  | ST-CA-ST / SC-CA-SC |  | 0.07 |
|  | ST-CA-ST / Nanogel |  | 0.66 |
|  | SC-CA-SC / Nanogel |  | 0.41 |
| Fig. 5B (6 weeks, SAG 21.99) | No enzymes / ST-CA-ST | Tukey HSD | 0.444 |
|  | No enzymes / SC-CA-SC |  | 0.088 |
|  | No enzymes / Nanogel |  | 1 |
|  | ST-CA-ST / SC-CA-SC |  | 0.006 |
|  | ST-CA-ST / Nanogel |  | 0.4 |
|  | SC-CA-SC / Nanogel |  | 0.101 |
| Fig. 5B (2 weeks, SAG 257.80) | No enzymes / ST-CA-ST | Dunnett T3 | 0.003 |
|  | No enzymes / SC-CA-SC |  | <0.001 |
|  | No enzymes / Nanogel |  | 0.248 |
|  | ST-CA-ST / SC-CA-SC |  | 0.197 |
|  | ST-CA-ST / Nanogel |  | 0.055 |
|  | SC-CA-SC / Nanogel |  | 0.015 |
| Fig. 5B (6 weeks, SAG 257.80) | No enzymes / ST-CA-ST | Tukey HSD | 0.999 |
|  | No enzymes / SC-CA-SC |  | 1 |
|  | No enzymes / Nanogel |  | 0.998 |
|  | ST-CA-ST / SC-CA-SC |  | 0.995 |
|  | ST-CA-ST / Nanogel |  | 0.989 |
|  | SC-CA-SC / Nanogel |  | 1 |
| Fig. 5C (2 weeks, SAG 21.99) | ST-CA-ST / SC-CA-SC | Tukey HSD | 0.909 |
|  | ST-CA-ST / Nanogel |  | 0.107 |
|  | SC-CA-SC / Nanogel |  | 0.056 |
| Fig. 5C (6 weeks, SAG 21.99) | ST-CA-ST / SC-CA-SC | Tukey HSD | 0.317 |
|  | ST-CA-ST / Nanogel |  | 0.036 |
|  | SC-CA-SC / Nanogel |  | 0.356 |
| Fig. 5C (2 weeks, SAG 257.80) | ST-CA-ST / SC-CA-SC | Tukey HSD | 0.48 |
|  | ST-CA-ST / Nanogel |  | 0.707 |
|  | SC-CA-SC / Nanogel |  | 0.872 |
| Fig. 5C (6 weeks, SAG 257.80) | ST-CA-ST / SC-CA-SC | Dunnett T3 | 0.723 |
|  | ST-CA-ST / Nanogel |  | 0.664 |
|  | SC-CA-SC / Nanogel |  | 1 |
| Fig. 6A (2 weeks) | No enzymes / ST-CA-ST | Dunnett T3 | <0.001 |
|  | No enzymes / SC-CA-SC |  | 0.001 |
|  | No enzymes / Nanogel |  | 0.013 |
|  | ST-CA-ST / SC-CA-SC |  | 0.053 |
|  | ST-CA-ST / Nanogel |  | 0.158 |
|  | SC-CA-SC / Nanogel |  | 0.699 |
| Fig. 6A (6 weeks) | No enzymes / ST-CA-ST | Dunnett T3 | 0.037 |
|  | No enzymes / SC-CA-SC |  | <0.001 |
|  | No enzymes / Nanogel |  | <0.001 |
|  | ST-CA-ST / SC-CA-SC |  | 0.859 |
|  | ST-CA-ST / Nanogel |  | 0.128 |
|  | SC-CA-SC / Nanogel |  | 0.002 |
| Fig. 6B (2 weeks) | No enzymes / ST-CA-ST | Dunnett T3 | 0.068 |
|  | No enzymes / SC-CA-SC |  | 0.005 |
|  | No enzymes / Nanogel |  | 0.075 |
|  | ST-CA-ST / SC-CA-SC |  | 0.936 |
|  | ST-CA-ST / Nanogel |  | 0.998 |
|  | SC-CA-SC / Nanogel |  | 0.775 |
| Fig. 6B (6 weeks) | No enzymes / ST-CA-ST | Tukey HSD | 0.088 |
|  | No enzymes / SC-CA-SC |  | 0.468 |
|  | No enzymes / Nanogel |  | 0.726 |
|  | ST-CA-ST / SC-CA-SC |  | 0.674 |
|  | ST-CA-ST / Nanogel |  | 0.42 |
|  | SC-CA-SC / Nanogel |  | 0.968 |
| Fig. 6D (2 weeks) | No enzymes / ST-CA-ST | Tukey HSD | 0.039 |
|  | No enzymes / SC-CA-SC |  | 0.014 |
|  | No enzymes / Nanogel |  | 0.105 |
|  | ST-CA-ST / SC-CA-SC |  | 0.999 |
|  | ST-CA-ST / Nanogel |  | 0.818 |
|  | SC-CA-SC / Nanogel |  | 0.686 |
| Fig. S10 | ST-CA-ST / SC-CA-SC | Dunnett T3 | 0.65 |
|  | ST-CA-ST / Nanogel |  | 0.023 |
|  | SC-CA-SC / Nanogel |  | 0.893 |
| Fig. S11C (2 mM) | ST-CA-ST / SC-CA-SC | Tukey HSD | 0.507 |
|  | ST-CA-ST / Nanogel |  | 0.207 |
|  | SC-CA-SC / Nanogel |  | 0.736 |
| Fig. S11C (4 mM) | ST-CA-ST / SC-CA-SC | Dunnett T3 | 0.91 |
|  | ST-CA-ST / Nanogel |  | 0.274 |
|  | SC-CA-SC / Nanogel |  | 0.5 |
| Fig. S11D (10 nM) | ST-CA-ST / SC-CA-SC | Tukey HSD | 0.945 |
|  | ST-CA-ST / Nanogel |  | 0.956 |
|  | SC-CA-SC / Nanogel |  | 0.819 |
| Fig. S11D (20 nM) | ST-CA-ST / SC-CA-SC | Tukey HSD | 0.316 |
|  | ST-CA-ST / Nanogel |  | 0.972 |
|  | SC-CA-SC / Nanogel |  | 0.42 |
| Fig. S11D (30 nM) | ST-CA-ST / SC-CA-SC | Tukey HSD | 0.889 |
|  | ST-CA-ST / Nanogel |  | 0.012 |
|  | SC-CA-SC / Nanogel |  | 0.024 |
| Fig. S11D (40 nM) | ST-CA-ST / SC-CA-SC | Tukey HSD | 0.324 |
|  | ST-CA-ST / Nanogel |  | 0.007 |
|  | SC-CA-SC / Nanogel |  | 0.059 |
| Fig. S12 (1 mg) | ST-CA-ST / SC-CA-SC | Tukey HSD | 0.982 |
|  | ST-CA-ST / Nanogel |  | 0.823 |
|  | SC-CA-SC / Nanogel |  | 0.724 |
| Fig. S12 (5 mg) | ST-CA-ST / SC-CA-SC | Dunnett T3 | 0.706 |
|  | ST-CA-ST / Nanogel |  | 0.5 |
|  | SC-CA-SC / Nanogel |  | 0.242 |

**References**

[1] M. G. d. Reis, A. Ribeiro, Conversion factors and general equations applied in agricultural and forest meteorology, Agrometeoros (2020) 27

[2] J. B. Vergnes, V. Gernigon, P. Guiraud, C. Formosa-Dague, Bicarbonate Concentration Induces Production of Exopolysaccharides by Arthrospira platensis That Mediate Bioflocculation and Enhance Flotation Harvesting Efficiency, ACS Sustainable Chemistry & Engineering (2019) Vol. 7, 13796-13804

[3] L. Trabelsi, N. H. M’sakni, H. Ben Ouada, H. Bacha, S. Roudesli, Partial characterization of extracellular polysaccharides produced by cyanobacterium Arthrospira platensis, Biotechnology and Bioprocess Engineering (2009), Vol. 14, 27-31
